# Exosomal miR-92a-3p promotes pancreatic cancer cells' extravasation by inducing vascular permeability through inhibition of DAB2IP

**DOI:** 10.1038/s41419-026-08719-9

**Published:** 2026-04-08

**Authors:** Luhan Li, Yanyan Cui, Miao Zhang, Tianyu Shen, Dekun Wang, Xue Mi, Yuying Zhang, Xiaoyue Tan, Alejandro Vaquero, Thomas Braun, Jihui Hao, Alessandro Ianni, Chunyang Jiang, Shijing Yue

**Affiliations:** 1 Department of Thoracic Surgery, Tianjin Union Medical Center, The First Affiliated Hospital of Nankai University, Nankai University, Tianjin, China; 2 School of Medicine, State Key Laboratory of Medicinal Chemical Biology, Nankai University, Tianjin, China; 3 The Affiliated Hospital of Chifeng University, Chifeng, China; 4 Department of Oncology, Tianjin Union Medical Center, The First Affiliated Hospital of Nankai University, Nankai University, Tianjin, China; 5 Chromatin Biology Laboratory, Josep Carreras Leukaemia Research Institute (IJC), Barcelona, Spain; 6 Department of Cardiac Development and Remodeling, Max-Planck-Institute for Heart and Lung Research, Bad Nauheim, Germany; 7 National Clinical Research Center for Cancer, Key Laboratory of Cancer Prevention and Therapy, Tianjin’s Clinical Research Center for Cancer, Department of Pancreatic Cancer, Tianjin Medical University Cancer Institute and Hospital, Tianjin, China

**Keywords:** Cancer microenvironment, Multivesicular bodies

## Abstract

Metastasis, the colonization of distant organs by cells derived from primary cancer, is the leading cause of mortality in pancreatic adenocarcinoma (PAAD). Growing evidence indicates that cancer-derived exosomes play pivotal roles in facilitating cancer metastasis by promoting pre-metastatic niche formation. However, the contribution of PAAD-derived exosomal microRNAs (MiRNAs) to this process remains poorly characterized. In this study, we identified specific PAAD-derived exosomal miRNAs involved in metastatic progression. Sequencing of small RNAs extracted from circulating exosomes derived from patients with metastatic or non-metastatic PAAD revealed that miR-92a-3p is associated with a metastatic phenotype. We demonstrated that exosomal miR-92a-3p facilitates cancer cells' extravasation and lung metastasis by disrupting vascular barrier integrity. Mechanistically, exosomal miR-92a-3p directly inhibits the tumor suppressor disabled homolog 2 interacting protein (DAB2IP), thereby activating the PI3K-AKT signaling cascade in endothelial cells (ECs). This activation attenuates expression of intracellular junction markers and stimulates endothelial nitric oxide synthase, leading to increased vascular permeability. Our findings suggest that targeting miR-92a-3p could represent a potential strategy to reduce metastasis in PAAD.

## Introduction

Pancreatic adenocarcinoma (PAAD) is a devastating malignancy that is often detected at a metastatic stage, with a five-year survival rate of only 13% [1, 2]. Approximately 85% of patients present unresectable cancer, and about 50% show metastases to the liver and lungs at the initial diagnosis [3]. Metastasis formation is a multistep process involving primary cancer outgrowth, local invasion, intravasation into blood vessels, extravasation of cancer cells at distant organs, survival of these cells within target tissues, and macroscopic metastatic growth [4]. Several studies have highlighted differences in aggressiveness and clinical outcomes among multi-organ metastatic lesions in PAAD [5, 6]. Although these investigations have characterized the spatial context of diverse cell populations in primary PAAD, the molecular mechanisms underlying metastatic biology remain elusive [7–9].

Over the past two decades, substantial evidence has established that the formation of a pre-metastatic niche (PMN) is essential for cancer cell dissemination and metastasis [10]. Numerous factors secreted by cancer cells contribute to this process [11–13]. Among them, exosomes appear to play pivotal roles in preparing distant tissues for the engraftment of cancer cells [13–18]. Exosomes are nanoscale vesicles (30–200 nm) secreted by virtually all cell types and participate in various biological processes. They carry diverse cargo molecules, including proteins, lipids, nucleic acids, and metabolites, and can influence target cells through multiple mechanisms. Proteins displayed on the exosomal surface can bind to specific cellular receptors, thereby triggering downstream signaling cascades. Additionally, exosomes are often internalized via endocytosis, releasing their cargos into recipient cells [19, 20].

Exosomes critically promote PMN establishment by altering endothelial properties to disrupt vascular integrity and facilitate cancer cell extravasation, remodeling the extracellular matrix and suppressing anti-cancer immune responses in target tissues [21–24]. Furthermore, molecules enriched in cancer-derived exosomes, such as integrins, appear to direct exosomes to specific organs, thereby conferring the ability of cancers to metastasize preferentially to certain sites, which is a phenomenon known as organotropism [25, 26]. Exosomes derived from PAAD cells are recognized as potent regulators of cancer progression, affecting proliferation, migration, and survival of neighboring cells in a paracrine manner [27–29]. Recent studies have also shown specific miRNAs to be enriched in circulating exosomes from PAAD patients compared to healthy controls [14, 30, 31]. Despite these findings, the role of PAAD-derived exosomal miRNAs in PMN formation remains poorly understood.

In this study, we identified miR-92a-3p as highly enriched in circulating exosomes from patients with metastatic PAAD. We demonstrate that exosomal miR-92a-3p promotes extravasation of PAAD cells in the lung by suppressing disabled homolog 2 interacting protein (DAB2IP) expression and disrupting intracellular junctions in endothelial cells (ECs), thereby increasing endothelial permeability. Mechanistically, exosomal miR-92a-3p activates the PI3K-AKT pathway in lung ECs via suppression of DAB2IP following uptake of PAAD-derived exosomes. Notably, administration of exosomes carrying miR-92a-3p antagomirs significantly reduced PAAD-derived metastases, suggesting a potential clinical application strategy for anti-cancer treatment. Lung metastasis is highly correlated with the liver but not the lymph node in the cohort of PAAD by statistical analysis. These findings indicate that exosomal miR-92a-3p supports PAAD lung and liver metastasis through the blood circulation.

## Results

### The miR-92a-3p is highly enriched in exosomes secreted by metastatic PAAD cells

**Fig. 1:**
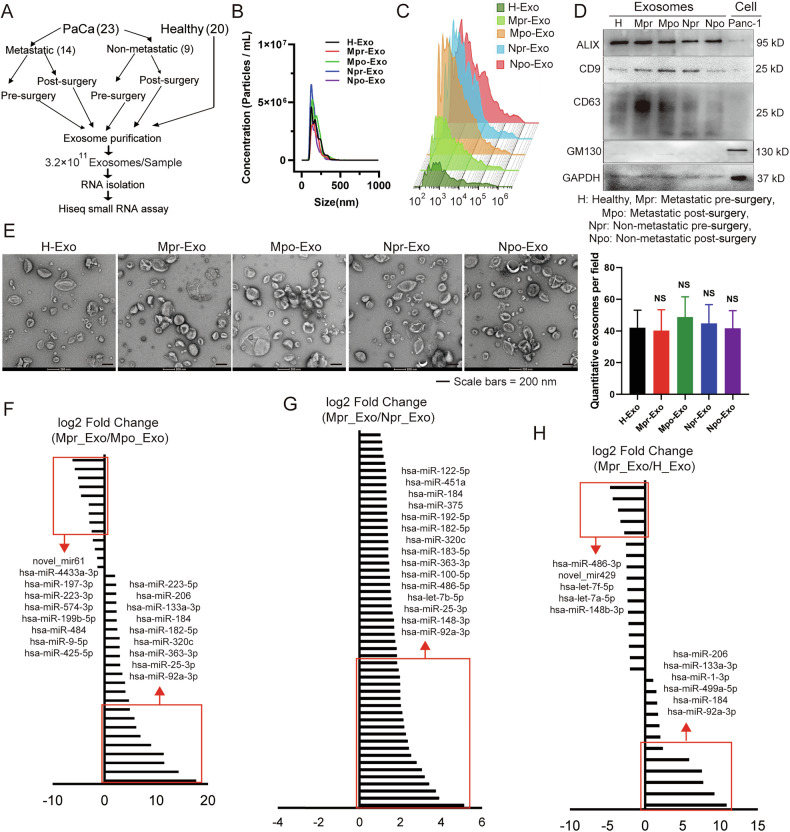
Exosomal miR-92a-3p is associated with a metastatic phenotype in PAAD. **A** Schematic overview of the strategy used to identify specific miRNAs associated with a metastatic phenotype in PAAD (plasma-derived exosomes, 3.2 × 10^11^ particles, were used for RNA preparation and sequencing). **B** Quantification and size distribution of exosomes analyzed by NTA. **C** Particle size distribution and concentration of isolated exosomes were also confirmed by NanoFCM. **D** Western blot analysis of exosomal markers (ALIX, CD63, and TSG101) and the Golgi marker GM130 in circulating exosomes from patients and healthy controls, confirming high purity of exosome preparations. Whole-cell lysate of Panc-1 cells served as a control. **E** TEM images of isolated exosomes showing characteristic morphology and size, and quantification of exosomes, one-way ANOVA, means ± SEM, *n* = 5 independent images, NS *P* ≥ 0.05. **F**–**H** Histograms showing the log2 fold change in expression of indicated miRNAs in exosomes derived from patients of different groups, as determined by miRNA sequencing. H Healthy, Mpr Metastatic pre-surgery, Mpo Metastatic post-surgery, Npr Non-metastatic pre-surgery, Npo Non-metastatic post-surgery.

To explore whether specific exosomal miRNAs are associated with a metastatic phenotype in human PAAD samples, we isolated circulating exosomes from the plasma of patients with either metastatic or non-metastatic PAAD. To ensure that exosomes originated from cancer cells, samples were collected from the same patients either before and one-week after cancer resection surgery. Isolated exosomes were subjected to small RNA sequencing, as outlined schematically (Fig. 1A). The study included a cohort of patients diagnosed with PAAD between 2015 and 2018 at the Tianjin Medical University Cancer Institute and Hospital (China). Patients’ characteristics are provided in Table. S1. The statistical correlation of different metastasis patterns was performed, which indicated liver and lung metastasis highly correlation (Tables. S2–S5). Twenty healthy donors were included as controls (Table. S6). Nanoparticle tracking analysis (NTA) was used to determine the particle size and concentration of the isolated exosomes were determined by NTA. The particle size distribution of the isolated exosomes. The size distribution ranged from 50 to 200 nm, with a peak at 146 nm (Fig. 1B). The particle size distribution of the isolated exosomes was also confirmed by NanoFCM (Fig. 1C). No significant difference in mean diameter of exosomes was observed among the different groups (Fig. S1A). Prior to sequencing, the purity of exosomes was assessed by western blot analysis of exosomal and non-exosomal markers. Exosomal markers ALIX, CD9, and CD63 were highly enriched in the exosome fractions compared to total cell lysates derived from Panc-1 cells, whereas GM130, a Golgi apparatus protein [32], was virtually absent in protein lysates from exosomal fractions, indicating high purity of preparation (Fig. 1D and Supplementary full blots). Transmission electron microscopy (TEM) confirmed that exosome morphology and size were consistent with established parameters [32]. To further confirm the purity of exosomes, the relative quantification of exosomes was performed by counting in TEM images. (Fig. 1E). Approximately 3.2 × 10^11^ plasma-derived exosomes were used to extract small RNAs for the miRNA Hiseq assay (Fig. 1A).

Exosomal miRNA Sequencing identified specific miRNAs expressed in each group: (1) 565 miRNAs in metastatic pre-operation patients (Mpr-Exo); (2) 474 miRNAs in metastatic post-operation patients (Mpo-Exo); (3) 435 miRNAs in non-metastatic pre-operation patients (Npr-Exo); (4) 496 miRNAs in non-metastatic post-operation patients (Npo-Exo); and 5) 670 miRNAs in healthy controls (H-Exo). Differential expression analysis was performed to identify miRNAs that varied between groups (Figs. 1F–H and S1B, C). The expression of certain miRNAs differed by more than 10 log2 fold in exosomes from patients before and after pancreatectomy (Mpr-Exo and Mpo-Exo, respectively), indicating that these miRNAs are enriched in PAAD cell-secreted exosomes (Fig. 1F). Among these, miR-92a-3p was the most highly abundant miRNA in circulating exosomes from metastatic patients before surgery (Mpr-Exo) compared with the post-operation stage (Mpo-Exo) (Fig. 1F). Notably, miR-92a-3p was also significantly enriched in metastatic patients compared with non-metastatic cancer patients (Npr-Exo) and to healthy controls (H-Exo) (Fig. 1G, H). These results show a cluster of miRNAs packaged in exosomes that is involved in cancer metastasis, including miR-25-3p, miR-363-3p, miR-320c, miR-182-5p, miR-183-5p and so on. Exosomes enriched the cluster of miRNAs for delivery to the target sites. Among the cluster of miRNAs, miR-92a-3p is the top one enriched in exosomes derived from metastatic cancer compared to non-metastatic cancer. In contrast, no significant change in exosomal miR-92a-3p was detected between non-metastatic PAAD patients before and after surgery or between non-metastatic patients and healthy controls (Fig. S1B, C). Taken together, these findings indicate that exosomal miR-92a-3p is closely associated with metastatic PAAD and may contribute to the formation of a pro-metastatic phenotype.

### PAAD cell-derived exosomes carrying miR-92a-3p reprogram EC phenotypes in vitro

**Fig. 2:**
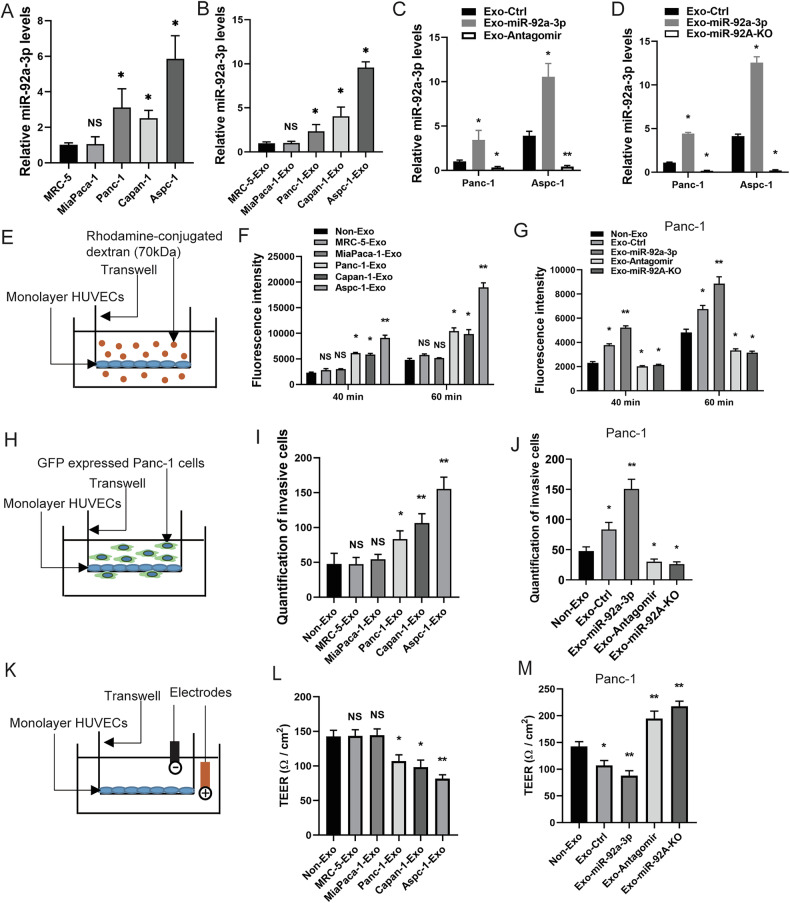
PAAD cell-derived exosomes enriched in miR-92a-3p increase EC permeability in vitro. **A** Relative miR-92a-3p levels in normal fibroblasts MRC-5 and PAAD cell lines (MiaPaca-1, Panc-1, Capan-1, Aspc-1) measured by stem-loop qPCR (Normalized to U6, U43, 18S). **B** Relative miR-92a-3p levels in exosomes derived from MRC-5 and PAAD cell lines. **C** Relative miR-92a-3p levels in exosomes from Panc-1 and Aspc-1 cells expressing miR-92a-3p or an Antagomir. **D** Relative miR-92a-3p levels in exosomes from Panc-1 and Aspc-1 cells expressing miR-92a-3p or with miR-92A KO. **E** Schematic of the permeability assay using HUVEC monolayers. Fluorescent rhodamine-dextran penetration through HUVEC monolayer pretreated with exosomes (1.8 × 10^11^ particles). **F**, **G** Quantification of fluorescence in the lower chamber medium, reflecting rhodamine-dextran penetration through the HUVEC monolayer treated with different exosomes. **H** Schematic of the transwell migration assay. Stable GFP-expressing Panc-1 cells were seeded on HUVEC monolayers pretreated with different PAAD cell-derived exosomes (1.8 × 10^11^ particles). **I**, **J** Quantification of transmigrated Panc-1 cells using ImageJ. **K** Schematic of the TEER measurement assay. TEER measurement of HUVEC monolayers was conducted pretreated with different PAAD cell-derived exosomes (1.8 × 10^11^ particles). **L**, **M** Quantification of TEER value of HUVEC monolayers. All quantifications present with one-way ANOVA, means ± SEM, *n* = 3, NS *P* ≥ 0.05, *P < 0.05, **P < 0.01.

Recent studies have shown that exosomes promote the PMN by inducing vascular barrier leakiness, thereby facilitating cancer cell extravasation into target tissues [21]. We hypothesized that exosomal miR-92a-3p may modulate the endothelium to promote cancer cell extravasation. To characterize the miRNA cargo of PAAD cell-derived exosomes, we first examined miR-92a-3p expression in fibroblast MRC-5 cells and PAAD cell lines (MiaPaca-1, Panc-1, Capan-1 and AsPc-1) by qPCR (Fig. 2A). MiR-92a-3p was up-regulated in Panc-1, Capan-1, and AsPc-1 cells but showed no significant difference in MiaPaca-1 cells compared with MRC-5 fibroblasts. qPCR analysis revealed that miR-92a-3p was highly enriched in exosomes derived from Panc-1, Capan-1 and AsPc-1 cells compared with those from MRC-5 cells (Fig. 2B). We next overexpressed miR-92a-3p or introduced a competitive inhibitor (Antagomir) and generated a miR-92A knockout (KO) in PAAD cell lines (Fig. S2). Exosomes were isolated from culture medium of Panc-1 cells expressing miR-92a-3p or Antagomir and characterized by TEM and western blot (Fig. S3A, B and Supplementary full blots). The abundance of miR-92a-3p in purified exosomes was then assessed. Exosomes from Panc-1 and Aspc-1 cells transfected with a miR-92a-3p expression plasmid contained high levels of miR-92a-3p, whereas a dramatic reduction was observed in exosomes from cells expressing the Antagomir or miR-92A KO cells (Fig. 2C, D).

To evaluate whether miR-92a-3p influences EC monolayer permeability in vitro, we treated human umbilical vein endothelial cell (HUVEC) monolayers in Transwell chambers with PAAD-derived exosomes enriched in miR-92a-3p, Antagomir or from miR-92A KO cells. Permeability was assessed by measuring the penetration of rhodamine-labeled dextran (70 kDa) through the HUVEC monolayer. We found that treatment with miR-92a-3p-enriched PAAD exosomes significantly increased EC permeability to fluorescent dextran compared with the non-exosome control. In contrast, exosomes with low miR-92a-3p content from MRC-5 fibroblasts or MiaPaca-1 cells showed no significant effect (Fig. 2E, F). Furthermore, exosomes from miR-92a-3p expressing Panc-1 and Aspc-1 cells significantly increased EC permeability, whereas exosomes from Antagomir expressing or miR-92A KO cells exerted an opposite effect (Figs. 2G and S4A). To determine whether exosomal miR-92a-3p induced EC permeability also facilitates PAAD cells transmigration, GFP-expressing Panc-1 cells were seeded on HUVEC monolayer pretreated with exosomes. Strikingly, we observed that ECs pretreated with miR-92a-3p-enriched PAAD exosomes allowed more efficient transmigration of PAAD cells through the endothelial monolayer compared with the non-exosome control (Fig. 2H, I). Moreover, exosomes from miR-92a-3p expressing Panc-1 and Aspc-1 cells significantly enhenced PAAD cell transmigration, whereas exosomes carrying Antagomir or derived from miR-92a-3p KO cells dramatically reduced it (Figs. 2J and S4B). In order to further evaluate exosomal miR-92a-3p impact on the integrity and permeability of the EC monolayer, we measured the barrier functions by trans-epithelial-endothelial electrical resistance (TEER). The barrier properties of the EC monolayer were assessed by TEER measurements after 3 days exosomes treatment. The low miR-92a-3p exosomes from MRC-5 fibroblasts and MiaPaca-1 cells did not show any significant change in TEER values compared with the non-exosome control. The most dominant permeability changes were observed with miR-92a-3p-enriched exosomes from Panc-1, Capan-1 and AsPc-1 cells (Fig. 2K, L). In addition, exosomes from miR-92a-3p expressing Panc-1 and Aspc-1 cells significantly decreased in TEER values, whereas exosomes carrying Antagomir or derived from miR-92a-3p KO cells dramatically increased it (Figs. 2M and S4C). These results demonstrate that PAAD-derived exosomal miR-92a-3p strongly increases endothelial permeability and facilitates PAAD cell transmigration in vitro.

### MiR-92a-3p induces vascular permeability by activating the PI3K-AKT pathway via inhibition of DAB2IP

**Fig. 3:**
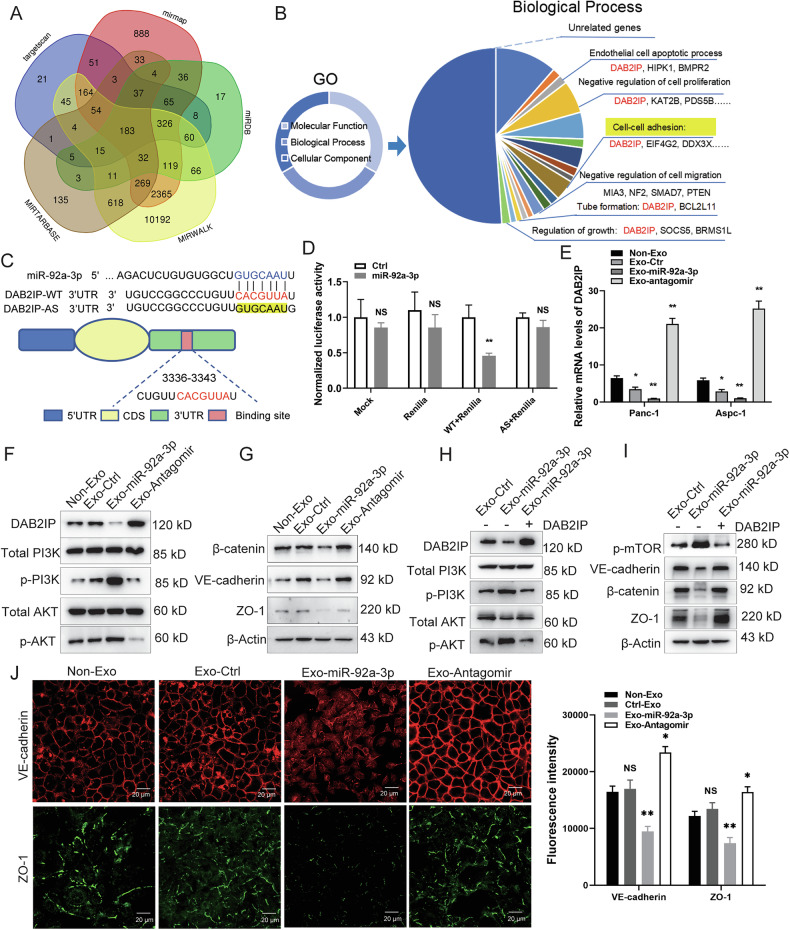
Exosomal miR-92a-3p targets DAB2IP, activates PI3K-AKT signaling, reduces cell-cell junctions, and promotes EC permeability. **A** Venn diagram showing common miR-92a-3p target genes predicted by the indicated databases. **B** GO analysis of the 183 common target genes, highlighting enrichment in cell adhesion, motility, and permeability pathways. **C** Schematic of WT and AS 3′ UTR sequences of DAB2IP containing the miRNA-92a-3p binding site. **D** Luciferase activity in 293T cells transfected with report constructs containing WT or AS 3′ UTR sequence of the DAB2IP, two-tailed Student’s *t* test, means ± SEM, *n* = 3, NS *P* ≥ 0.05, **P < 0.01. **E** Relative mRNA levels of DAB2IP in HUVECs treated with exosomes enriched miRNA-92a-3p, Antagomir, or control miRNA, as assessed by RT-qPCR, one-way ANOVA, means ± SEM, *n* = 3, *P < 0.05, **P < 0.01. **F**–**I** Western blot analysis of PI3K-AKT pathway markers and junctional proteins in HUVECs treated with different exosomes (1.8 × 10^11^ particles), or overexpressing DAB2IP for functional rescue assay. **J** IF staining of VE-cadherin (red), ZO-1 (green) in HUVECs treated with different exosomes (1.8 × 1011 particles). Fluorescence intensity was quantified using ImageJ, one-way ANOVA, means ± SEM, *n* = 3, NS *P* ≥ 0.05, *P < 0.05, **P < 0.01.

To elucidate the molecular mechanisms by which the exosomal miR-92a-3p regulates the endothelium, we used bioinformatic tools (TargetScan, miRDB, Mirmap, MIRWALK, and MIRTARBASE) to identify potential target genes and performed Gene Ontology analysis. Potential miR-92a-3p targets are involved in biological processes such as cell–cell adhesion, migration, permeability and proliferation (Fig. 3A, B). The DAB2IP gene emerged as an attractive candidate, given its involvement in processes contributing to metastases. In silico analysis confirmed the presence of a miR-92a-3p binding site in the 3′UTR of DAB2IP mRNA (position: 3336-3343; GUGCAAUU). To validate DAB2IP as a direct target, we generated luciferase reporter constructs (Fig. 3C). Wild-type (WT) and antisense (AS) 3´UTR sequences of DAB2IP were cloned downstream of the luciferase cDNA, and these constructs were transfected alone or together with miR-92a-3p or a control miRNA into 293T cells. Luciferase assays showed that miR-92a-3p effectively reduced expression from the WT but not the AS constructs (Fig. 3D). To further confirm that DAB2IP is a direct target of exosomal miR-92a-3p, HUVECs were treated with PAAD cell-derived exosomes, and DAB2IP expression was assessed at the mRNA and protein levels (Fig. 3E, F). We found that miR-92a-3p-enriched exosomes dramatically inhibited DAB2IP expression in ECs, whereas the Antagomir increased DAB2IP RNA and protein levels (Fig. 3E, F). These data indicate that PAAD exosomal miR-92a-3p reduces DAB2IP expression in ECs.

DAB2IP, a member of the Ras GTPase-activating protein family, is highly expressed in ECs and regulates inflammation and apoptosis [33]. Interestingly, DAB2IP acts as a potent inhibitor of the PI3K-AKT pathway in prostate cancer cells by interacting with the PI3K regulatory subunit p85 [34], an interaction we also detected in ECs (Fig. S5A and Supplementary full blots). The PI3K-AKT pathway plays critical roles in EC physiology, promoting proliferation and microvascular permeability by stimulating endothelial nitric oxide synthase (eNOS) activity and nitric oxide production [35]. Knockdown of DAB2IP in ECs confirmed that DAB2IP inhibits PI3K-AKT activation and eNOS expression (Fig. S5B and Supplementary full blots). Consistent with DAB2IP’s inhibitory roles, its suppression reduced expression of the endothelial junction molecules occludin, VE-cadherin, and β-catenin (Fig. S5C and Supplementary full blots). These findings are in line with previous studies that PI3K-AKT activation compromises vascular barrier integrity by downregulating genes required for adherens junctions (AJs) and tight junctions (TJs) [21, 36].

To corroborate the hypothesis that exosomal miR-92a-3p increases vascular permeability by suppressing DAB2IP, we analyzed the effects of miR-92a-3p-enriched PAAD exosomes on ECs. We found that exosomal miR-92a-3p enhanced activation of the PI3K-AKT cascade, as indicated by increased phosphorylation of AKT and PI3K, and reduced expression of the junction molecules β-catenin, VE-cadherin, and ZO-1 (Fig. 3F, G, and Supplementary full blots). To prove DAB2IP is the critical downstream mediator of exosomal miR-92a-3p, we performed a rescue experiment by expressing DAB2IP without the 3′UTR sequence in ECs. DAB2IP is stably expressed in ECs with miR-92a-3p-enriched PAAD exosome treatment (Fig. 3H). We found that DAB2IP expression effectively reversed exosomal miR-92a-3p induced the activation of the PI3K-AKT pathway and restored expression of the junction molecules β-catenin, VE-cadherin, and ZO-1 (Fig. 3H, I, and Supplementary full blots). DAB2IP expression significantly rescued exosomal miR-92a-3p induced EC permeability. PI3K-AKT inhibitor LY294002 treatment also rescued exosomal miR-92a-3p induced EC permeability (Fig. S5D, E). Transwell assay demonstrated that expressing miR-92a-3p promoting PAAD cell motility and KO miR-92A inhibits it (Fig. S5F). Immunofluorescence (IF) analysis further confirmed that miR-92a-3p-enriched exosomes strongly decreased levels of VE-cadherin and ZO-1, whereas Antagomir-containing exosomes exhibited opposite effects (Fig. 3J). Together, these data provide compelling evidence that exosomal miR-92a-3p downregulates DAB2IP to activate the PI3K-AKT pathway, ultimately leading to increased endothelial permeability.

### Exosomal miR-92a-3p is taken up by lung ECs and impairs vascular integrity in vivo

**Fig. 4:**
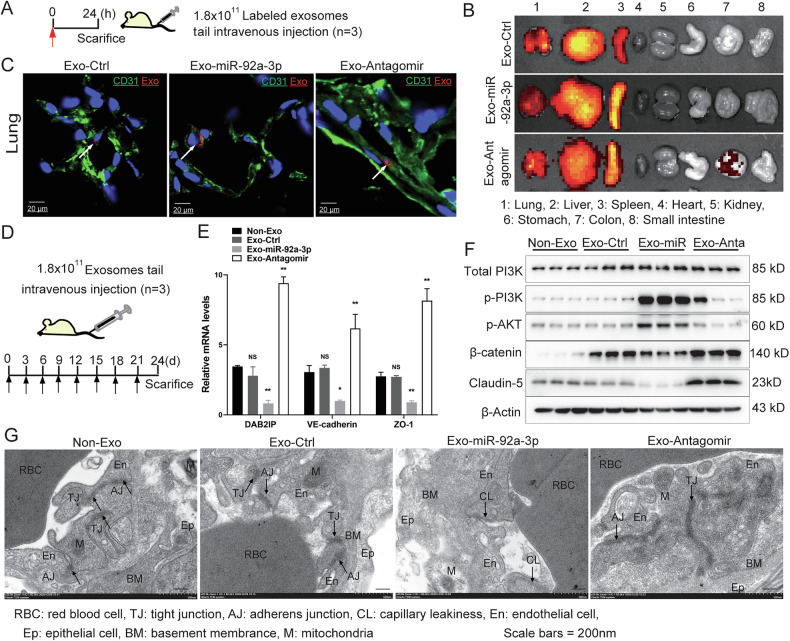
Exosomal miR-92a-3p induces vascular leakiness in lungs. **A** Schematic of the experimental design for tracking PAAD cell-derived exosome distribution in vivo following tail-vein injection (1.8 × 10^11^ particles). **B** Whole‑mount imaging showing biodistribution of PAAD cell-derived exosomes in vivo. **C** IF staining of CD31 (green) and PAAD cell-derived exosomes (red) in lung capillaries. **D** Schematic of the procedure used to assess the effect of exosomes on vascular barrier integrity in vivo (tail‑vein injection, 1.8 × 10^11^ particles). **E** Relative mRNA levels of DAB2IP, VE-cadherin, and ZO-1 measured by qPCR in ECs isolated from the lung tissues of exosome-treated mice, one-way ANOVA, means ± SEM, *n* = 3, NS *P* ≥ 0.05, *P < 0.05, **P < 0.01. **F** Western blot analysis of indicated markers in lung ECs from exosome-treated mice. **G** Representative TEM images of lung sections from the mice treated with exosomes.

We next investigate the role of the exosomal miR-92a-3p-DAB2IP axis in endothelial barrier integrity and cancer cell extravasation in vivo. Firstly, the distribution of PAAD-derived miR-92a-3p-enriched exosomes was examined. Fluorescent DiR-labeled exosomes were injected into nude mice, and whole-mount imaging of organs was performed after 24 h (Fig. 4A, B). These experiments revealed that Panc-1-derived exosomes predominantly accumulated in the lung, liver, and spleen. IF analysis of lung tissue using CD31 antibody further confirmed that lung ECs effectively internalized exosomes derived from PAAD cells, irrespective of miR-92a-3p or Antagomir expression (Fig. 4C). These findings indicate that PAAD-derived exosomes preferentially accumulate in lung tissue and are partly taken up by ECs.

The strong accumulation of PAAD-derived exosomes in the lung, which is one of the major sites of PAAD metastasis [37, 38], prompted us to investigate the effect of exosomal miR-92a-3p on endothelial integrity in this organ. Mice were administered miR-92a-3p-enriched, antagomir-containing, or control exosomes every 3 days for 3 weeks (Fig. 4D). ECs were subsequently isolated from lung tissue by flow cytometry to assess DAB2IP expression and PI3K-AKT pathway activation. qPCR analysis showed that treatment with miR-92a-3p-enriched PAAD exosomes reduced mRNA levels of DAB2IP, E-cadherin and ZO-1 in lung ECs, while Antagomir-containing exosomes increased their expression (Fig. 4E). Western blot analysis revealed elevated phosphorylation of PI3K and AKT in lung ECs from mice injected with miR-92a-3p-enriched exosomes, whereas Antagomir exosomes reduced PI3K and AKT activation (Fig. 4F and Supplementary full blots). Consistent with in vitro observations, miR-92a-3p-enriched exosomes correlated with increased PI3K-AKT signaling and reduced expression of β-catenin and claudin-5, whereas Antagomir exosomes exerted opposite effects (Fig. 4F). These findings revealed that PAAD exosomal miR-92a-3p enhances PI3K-AKT pathway activity and downregulates endothelial junction molecules in the lung.

To further evaluate the role of exosomal miR-92a-3p in endothelial barrier disruption, we performed TEM of lung endothelium (Fig. 4G). The analysis revealed well-defined TJs and AJs. Given that loss of vascular integrity may facilitate cancer cell extravasation and metastasis, we next examined the effect of miR-92a-3p-enriched exosomes on PAAD cell colonization in the lung.

### Exosomal miR-92a-3p promotes PAAD lung metastasis in vivo

**Fig. 5:**
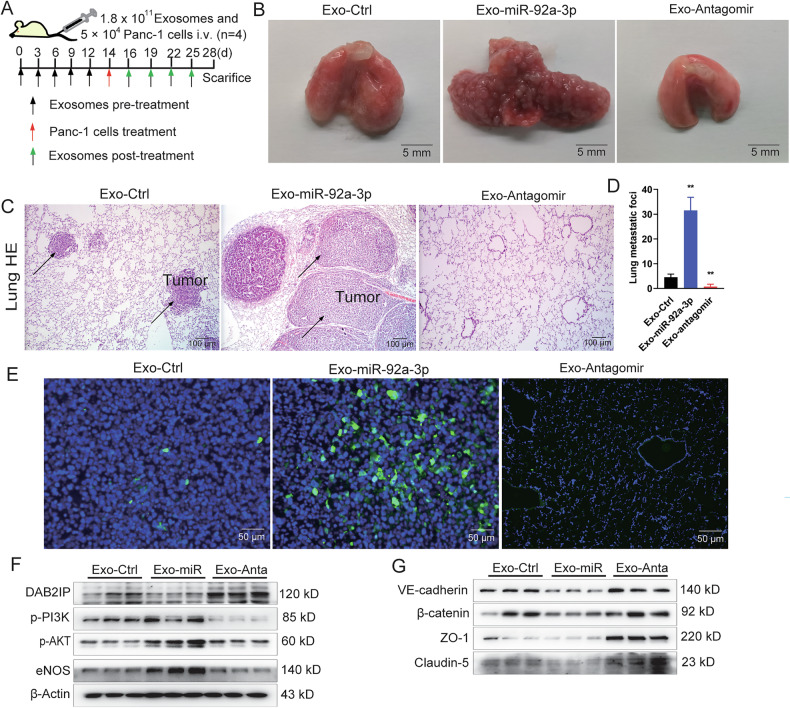
Exosomal miR-92a-3p promotes PAAD lung metastasis formation. **A** Schematic of the experimental design to evaluate the effect of PAAD cell-derived exosomes on cancer cell extravasation in vivo. Exosomes were administered via tail‑vein injection (1.8 × 10^11^ particles). **B** Macroscopic picture of lung tissues from mice treated with exosomes and PAAD cells. **C** H&E staining of lung tissues derived from mice treated with exosomes and PAAD cells. **D** Quantification of PAAD lung metastasis foci formation in mice treated with exosomes and PAAD cells, one-way ANOVA, means ± SEM, *n* = 4, **P < 0.01. **E** IF detection of GFP-expressing Panc-1 cells in the tumor-bearing and tumor-free lung tissues. **F**, **G** Western blot analysis of PI3K‑AKT pathway markers and junctional proteins in lung tissues containing tumor foci.

To determine whether exosomal miR-92a-3p promotes cancer cell extravasation in vivo, nude mice were pretreated with exosomes for 2 weeks, followed by intravenous injection of 1 × 10^6^ Panc-1 cells and continued exosome treatment as outlined in Fig. 5A. qPCR analysis confirmed significant accumulation of miR-92a-3p in the lungs of mice treated with miR-92a-3p-enriched exosomes and reduced levels in those receiving Antagomir exosomes (Fig. S6A). Macroscopic examination, Hematoxylin and eosin (H&E), and IF staining of lung sections showed that mice treated with miR-92a-3p-enriched exosomes developed significantly more PAAD-derived lung cancers (Figs. 5B–E and S6B) and exhibited greater body weight loss compared with controls (Fig. S6C). Notably, mice receiving Antagomir exosomes were protected from lung metastatic cancer formation and body weight loss (Figs. 5B–E and S6C). Western blot analysis further confirmed enhanced PI3K-AKT pathway activation and reduced expression of the vascular junction molecules in lung tissues from the miR-92a-3p group (Fig. 5F, G, and Supplementary full blots). These results indicate that sustained treatment with miR-92a-3p-enriched exosomes enhances the lung colonization by PAAD cells, whereas Antagomir exosomes block this effect. Mechanistically, miR-92a-3p exosomes downregulated DAB2IP, upregulated phospho-PI3K, phospho-AKT, and eNOS, and reduced the vascular junction protein expression, whereas Antagomir exosomes exerted opposite effects.

### Deletion of miR-92A in PAAD cells impairs lung metastasis

**Fig. 6:**
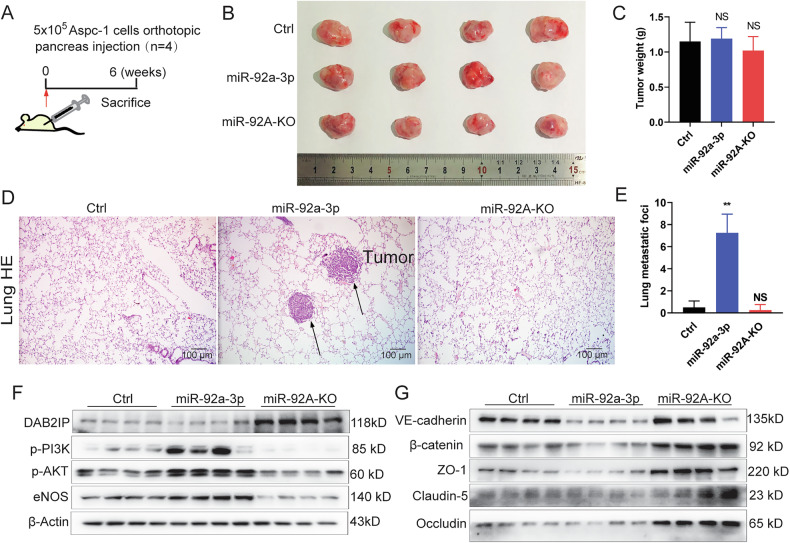
Expression of miR-92a-3p promotes PAAD metastasis. **A** Schematic of the experimental design to assess the role of miR-92a-3p on PAAD development. **B** Macroscopic images of primary tumors in orthotopic PAAD mouse models. **C** Expressing miR-92a-3p or KO of miR-92A did not impact on the growth of primary tumor, one-way ANOVA, means ± SEM, *n* = 4, NS *P* ≥ 0.05. **D** H&E staining of lung tissues with or without metastatic lesions in orthotopic PAAD mouse models. **E** Quantification of lung metastasis foci formation in orthotopic PAAD mouse models, one-way ANOVA, means ± SEM, *n* = 4, NS *P* ≥ 0.05, **P < 0.01. **F**, **G** Western blot analysis of PI3K‑AKT pathway markers and junctional proteins in lung tissues from orthotopic PAAD mouse models.

To further characterize the miR-92a-3p-DAB2IP axis in vivo, we established an orthotopic PAAD model using control, miR-92a-3p-expressing, and miR-92A KO Aspc-1 cells in nude mouse (Fig. 6A). Primary cancer size and weight did not differ significantly among groups (Fig. 6B, C). However, lung metastases assessment revealed a significant increase in metastatic foci in the miR-92a-3p group and a decrease in the KO group, as shown by H&E staining (Fig. 6D) and macroscopic quantification (Fig. 6E). Western blot analysis of orthotopic cancer tissues showed reduced DAB2IP protein levels in miR-92a-3p group and increased levels in the KO group (Fig. 6F, G, and Supplementary full blots). Consistent with data obtained in vitro, PI3K-AKT pathway activation and eNOS expression were elevated in the miR-92a-3p group and reduced in the KO group (Fig. 6F). Expression of E-cadherin, β-catenin, ZO-1, Claudin-5 and Occludin was reduced in the miR-92a-3p group and increased in the KO group (Fig. 6G). These findings suggest that miR-92a-3p activates the PI3K-AKT pathway and downregulates intracellular junction molecules in PAAD cells, thereby enhancing their detachment from the primary cancer, which is a key step in metastasis. Exosomes from miR-92a-3p-overexpressing PAAD cells are taken up by lung ECs and promote vascular leakiness, facilitating lung metastasis. Conversely, miR-92A KO in PAAD cells inhibits metastasis in vivo.

## Conclusion

Collectively, our findings demonstrate that miR-92a-3p expression in PAAD cells plays a critical role in metastasis. By inhibiting DAB2IP, miR-92a-3p activates the PI3K-AKT pathway and reduces intercellular adhesion in PAAD cells, promoting their motility and detachment from the primary site. Exosomes enriched with miR-92a-3p accumulate in lung tissue and are internalized by ECs. Through suppression of DAB2IP in ECs, exosomal miR-92a-3p activates PI3K-AKT signaling, increases vascular permeability, and supports PAAD cell extravasation, thereby driving lung metastasis.

## Discussion

PAAD is a highly aggressive malignancy with a dismal prognosis, marked by a 5-year survival rate of only 13% and a median survival of 5-8 months [3]. The absence of reliable biomarkers for early detection often results in diagnosis at a metastatic stage. While surgical resection is considered curative, approximately 20% of patients who undergo resection still succumb to the disease due to local and distant recurrence [39, 40]. Therefore, elucidating the molecular mechanisms that drive PAAD metastasis is crucial for developing novel clinical interventions.

Cancer cell-derived exosomes are increasingly recognized as key mediators of cancer progression, functioning through autocrine, paracrine, and endocrine signaling [37, 38]. Notably, exosomes contribute to the formation of a PMN in distant tissues [25, 41]. Compelling evidence indicates that exosomes from highly aggressive PAAD cells enhance the proliferation of less malignant cells and promote migration and chemoresistance by activating detoxification and anti-apoptotic pathways [13, 42, 43]. A recent study showed that PAAD-derived exosomes stimulate hepatic fibronectin secretion, recruiting bone marrow-derived macrophages to facilitate PMN establishment and liver metastasis [13]. Despite these advances, the mechanisms by which exosomes promote PAAD metastasis remain poorly understood.

As major RNA cargo within exosomes, miRNAs have garnered significant attention. Exosomal miRNAs mediate novel interactions among Cholangiocarcinoma (CCA) cells, mast cells (MCs) and bile. For example, bile-derived exosomes enriched in miR-182/183-5p inhibit hydroxyprostaglandin dehydrogenase in CCA cells and MCs, increasing prostaglandin E2 and VEGF-A release [44]. Additionally, miR-320c has been identified as both a biomarker for oxaliplatin responsiveness and a therapeutic target to enhance chemotherapy efficacy in triple-negative breast cancer [45]. Drobna et al. demonstrated that hsa-miR-20b-5p and hsa-miR-363-3p from the mir-106a-363 cluster act as oncomiRs in T-cell acute lymphoblastic leukemia by post-transcriptionally repressing the tumor suppressors PTEN and BIM [46]. Similarly, liposarcoma (LPS)-derived exosomes carrying miR-25–3p and miR-92a-3p stimulated IL-6 secretion from tumor-associated macrophages, promoting LPS metastasis [47]. Disruption of vascular barrier integrity is a critical step in cancer cell extravasation and subsequent metastasis [41]. Various factors secreted by or expressed on cancer cells contribute to this process by inducing EC remodeling or death [48, 49]. Evidence from other cancers demonstrates that exosomal miRNAs from cancer cells play a pivotal role in reducing vascular integrity to facilitate metastasis [50], although other exosomal cargoes also contribute [51].

In this study, we identified miR-92a-3p as highly enriched in exosomes secreted by metastatic PAAD cells. Our results are in line with previous reports linking elevated circulating exosomal miR-92a-3p levels to poor patient prognosis [47, 52]. In mice, PAAD-derived exosomes containing miR-92a-3p accumulate in the lung and liver, the primary sites of PAAD metastasis, as well as in the spleen [2, 7]. In the lung, exosomal miR-92a-3p activates the PI3K-AKT pathway by suppressing DAB2IP, a known inhibitor of this axis [35]. This leads to increased eNOS production and reduced expression of key endothelial junction proteins, resulting in vascular leakiness [38]. Such barrier disruption facilitates PAAD cell extravasation and metastases. Notably, Neutralizing miR-92a-3p with an Antagomir significantly reduced PAAD metastatic capacity in the lung. We propose that exosome-delivery miR-92a-3p Antagomir could counteract PAAD-derived miR-92a-3p, preventing DAB2IP suppression and subsequent PI3K-AKT activation. Alternatively, directly inhibiting PI3K-AKT signaling in ECs may represent another strategy to stabilize vessels and block cancer cell extravasation. Additionally, miR-92a-3p enhances PI3K-AKT signaling and impairs cell-cell adhesion in PAAD cells, thereby promoting motility. Consistent with this, our in vivo data demonstrate that miR-92A KO markedly suppresses PAAD metastasis. These data indicate that DAB2IP suppression effectively activates the PI3K-AKT pathway in ECs in vitro. However, PAAD-derived exosomes containing a cluster of miRNAs promote cancer metastasis by impacting cancer cells, ECs, and the distant microenvironment in vivo. DAB2IP knockdown alone in ECs is not sufficient to change cancer metastasis in vivo. Furthermore, the statistical analysis of the PAAD cohort showed that Lung metastasis is highly correlated with the liver but not the lymph node. These results demonstrated that exosomal miR-92a-3p supports PAAD distant metastasis through the blood circulation.

Beyond vascular permeability, exosomal miR-92a-3p from PAAD cells may regulate additional prometastatic mechanisms. For instance, miR-92a-3p is highly expressed in exosomes from metastatic hepatocellular carcinoma cells, where it induces epithelial-to-mesenchymal transition in low metastatic potential cells to promote migration and metastasis [53]. Elevated circulating exosomal miR-92a-3p has been correlated with worse prognosis in patients [47]. Moreover, cancer-associated fibroblasts-derived exosomes enriched in miR-92a-3p enhance metastasis, stemness, and drug resistance in colorectal cancer by activating the Wnt/β-catenin pathway [54]. Exosomes have recently emerged as a novel modality for cancer treatment due to their ease of engineering and low immunogenicity. We propose that administration of exosomes carrying miR-92a-3p Antagomir represents a potential new strategy for clinical interventions in metastatic PAAD. Further research is needed to support this hypothesis.

## Materials and methods

### Cell lines and cell culture

Human PAAD cell lines MiaPaca-1, Panc-1, Capan-1, and AsPc-1 were provided by Prof. Margot Zoller (University of Heidelberg, Germany) [55]. 293 T, HUVECs and human fetal lung fibroblasts MRC-5 were purchased from the American Type Culture Collection. MiaPaca-1, Panc-1, Capan-1 and AsPc-1 cells were cultivated in RPMI-1640 medium (Gibco, Thermo Fisher Scientific, San Jose, CA, USA) supplemented with 10% fetal bovine serum (FBS) (Gibco), non-essential amino acids (Gibco), 10 mM sodium pyruvate and 1% penicillin/streptomycin (Gibco). 293T and MRC-5 cells were grown in complete Dulbecco’s modified Eagle’s medium (Gibco) supplemented as above. HUVECs were maintained in complete EC M199 growth medium (Gibco) supplemented with 10% FBS, 0.1 mg/mL heparin, 30 *µ*g/mL EC growth supplement (Gibco) and 1% penicillin/streptomycin. All cell lines were maintained in a humidified incubator at 37 °C with 5% CO_2_.

### Generation of PAAD cell expressing miR-92a-3p, Antagomir, or DAB2IP, and knockdown lines

Lentiviral particles encoding mature miR-92a-3p, Antagomir, and control miRNA were obtained from GenePharma (Shanghai, China). Stable PAAD cell lines (Panc-1 and Aspc-1) were generated by lentiviral infection in the presence of 8 μg/mL polybrene. The culture medium was replaced after 48 h. Infected cells were selected with puromycin (1 µg/mL) to obtain stable expression of miR-92a-3p and Antagomir. The cDNA of DAB2IP was inserted into the lentiviral expression vector pLV-EF1α-MCS-IRES-Bsd/puro (Biosettia, San Diego, CA, USA). Stable DAB2IP knockdown cell lines were generated using lentiviral particles containing gene-targeting shRNAs as described previously [56], by cloning specific shRNAs into the pLV-H1-RNAi-Vector (Biosettia). All the viral experiments were performed in a biological safety cabinet. Oligonucleotide sequences of miRNAs and shRNAs used in this study are listed in Tables. S7 and S8, respectively.

### Generation and characterization of miR-92A KO PAAD cell lines

To investigate the function of miR-92a-3p in PAAD metastasis, the genomic regions encoding the mature miR-92a-3p were deleted in the PAAD cell lines Panc-1 and Aspc-1 using a CRISPR-Cas9 approach. Single-guide RNAs (sgRNAs) targeting sequence regions flanking the precursor miR-92A1 and miR-92A2 loci are shown in Supplementary Fig. S2. Double KO of miR-92A1 and miR-92A2 was performed sequentially. Briefly, two sgRNAs for miR-92A1 (GTTACTGAACACTGTTCTATGG, GTATCTTGTACATTTAACAGTGG) and two sgRNAs for miR-92A2 (ATGCAACAAATCCCCACCCAGGG, ATAAAGTATTGCACTTGTCCCGG) were synthesized and cloned into the pX458 vector containing EGFP to enable single-cell sorting. Panc-1 and Aspc-1 cells were co-transfected with plasmids px458-sgRNA1 and px458-sgRNA2 of miR-92A1 using Lipofectamine 3000 reagent in Opti-MEM medium. Single-cell sorting was performed 48 h post-transfection using the BD FACSAria™ Fusion system. Individual colonies derived from the single cells were screened by PCR to verify miR-92A1 KO. The same protocol was then applied to miR-92A1 KO cells to generate miR-92A2 KO. Oligonucleotide sequences of the guide RNAs and the genotyping primers for miR-92A1 and miR-92A2 KO are listed in Tables. S9 and S10, respectively. A schematic of the miR-92A1 and miR-92A2 KO strategy is shown in S2A, S2D and genotyping was confirmed by PCR (Figs. S2B, C and S2E, F).

### Human samples

Blood samples were collected from 23 patients with PAAD and 20 healthy donors. All participants provided signed informed consent. The study was approved by the Ethics Committee of Nankai University (Approval No. NKOIRB2021005). Patients and healthy donors information is provided in Tables. S1 and S6, respectively.

### Isolation of exosomes

Exosomes were purified from plasma or cell culture supernatants as previously described [57, 58]. Briefly, exosomes were isolated based on size and density using sequential centrifugation. For plasma-derived exosomes, blood was collected in EDTA-containing tubes and centrifuged twice at 2500 × *g* for 15 min to obtain the plasma [57]. Plasma was centrifuged at 10,000 × *g* for 30 min to remove large extracellular vesicles, followed by 100,000 × *g* for 90 min to pellet exosomes. Exosomes were washed in phosphate-buffered saline (PBS) and collected by centrifugation at 100,000 × *g* for 90 min [57]. For cell culture-derived exosomes, supernatants were sequentially centrifuged at 500 × *g* for 10 min (twice), 2000 × *g* for 20 min, and 12,000 × *g* for 30 min. The supernatant was filtered through a 0.22 µm pore size membrane and centrifuged at 100,000 × *g* for 90 min to pellet exosomes. Exosomes were washed in PBS and collected by centrifugation at 100,000 × *g* for 90 min.

### Characterization of exosomes

Exosome morphology and purity were assessed by TEM. Exosomes were resuspended in 2% paraformaldehyde and adsorbed onto carbon-coated formvar EM grids for 20 min. Grids were washed in physiological saline, incubated in 50 mM glycine/PBS for 3 min (repeated three times), embedded in 30 μL uranyl-oxalate solution for 90 s, and air-dried. Images were captured using an FEI Talos F200C TEM (Thermo Fisher Scientific). Exosomes' size and concentration were measured using a NanoSight NS300 NTA device (Malvern, UK). Protein concentration was determined with a bicinchoninic acid (BCA) assay kit (Pierce, Thermo Fisher Scientific). Based on NTA and BCA measurements, the particle-to-protein ratio of plasma-derived exosomes was approximately 1.6 × 10^10^ Particles/*μ*g, and that of cell culture supernatant-derived exosomes was approximately 1.8 × 10^10^ Particles/*μ*g. These ratios and the TEM images indicated high purity of exosomes. For further purity assessment, exosomes were quantified by counting in TEM images. Exosome particles were also analyzed using a flow nanoanalyser instrument manufactured by nanoFCM (U30, NanoFCM Inc, Xiamen, China). Silica nanospheres (250 nm diameter) were used for optical calibration and as a concentration standard. Samples were acquired at a sampling pressure of 1.0 kPa for 1 min. Size distribution and particle concentration data were analyzed with NanoFCM Software V2.31 (NanoFCM Inc.).

### miRNA, mRNA extraction, and real-time quantitative PCR (qPCR) analysis

Exosomal miRNAs were extracted using RNeasy mini spin columns (QIAGEN, Hilden, Germany) according to the manufacturer’s instructions. Exosome normalization was based on protein content and particle concentration. Small RNAs were extracted from approximately 3.2 × 10^11^ plasma-derived exosomes using the miRNeasy serum/plasma micro kit (QIAGEN). Small RNA concentration and quality were assessed using an Agilent 2100 Bioanalyzer and Agilent RNA 6000 Nano Kit (Agilent Technologies, Santa Clara, CA, USA). Sequencing was performed on an Illumina HiSeq 2500 system (BGI, Shenzhen, China). Raw sequencing data are available in the NCBI Gene Expression Omnibus under accession number GSE166799.

Total RNA from cells and tissues was extracted with TRIzol reagent (Invitrogen, Thermo Fisher Scientific) according to the manufacturer’s instructions. cDNA was synthesized using Trans Script First-Strand cDNA Synthesis Super Mix Kit (Trans Gen Biotech, Beijing, China). qPCR was performed with Trans Start Top Green qPCR Super Mix (Trans Gen Biotech) on a CFXTM real-time thermal cycler (Bio-Rad, Hercules, CA, USA). miRNA expression was measured by stem-loop qPCR as described previously [31]. Data were analyzed using the comparative ΔCt method, with normalization to U6, U43 small RNAs, or 18S ribosomal RNA [31]. Primer sequences are listed in Tables. S11 and S12.

### Dual-luciferase reporter assays

The effect of miR-92a-3p on DAB2IP expression was evaluated using a dual-luciferase reporter gene assay kit (YEASEN, Shanghai, China). WT and AS 3′UTR sequences of DAB2IP were inserted into the pGL3 vector (Promega, Beijing, China). Constructs were transfected into 293 T cells with a control miRNA or miR-92a-3p plasmid. Luciferase activity was measured 48 hours post-transfection using an Infinite M200 PRO microplate reader (TECAN, Männedorf, Switzerland). Primer sequences for vector construction are listed in Table. S13.

### Western blot analysis

Western blotting was performed as previously described [56]. Proteins were lysed in radioimmunoprecipitation assay buffer (25 mM Tris-HCl pH 7.6, 150 mM NaCl, 1% sodium deoxycholate, 0.1% SDS) supplemented with protease inhibitor cocktail (Sigma, MO, USA). Protein concentration was determined with a BCA assay kit (Pierce). Proteins were separated by sodium dodecyl sulphate-polyacrylamide gel electrophoresis (SDS-PAGE) and transferred onto polyvinylidene difluoride membranes. Membranes were blocked with 5% milk in Tris-buffered saline (TBS) containing 0.1% Tween 20 (TBS-T) for 1 h at 20 °C, then incubated overnight at 4 °C with primary antibodies. After washing, membranes were incubated with HRP-conjugated secondary antibodies for 1 h at 20 °C. Signals were detected using a Tanon Chemiluminescent Imaging System (Tanon, Shanghai, China). Antibodies are listed in Table. S14.

### Permeability of HUVECs treated with exosomes

HUVEC monolayer permeability was assessed using a Transwell assay. HUVECs were pre-treated with exosomes (1.8 × 10^11^ Particles/mL) for two passages and seeded on Transwell inserts (4 µm pore size) to generate a monolayer. Rhodamine-labeled dextran probe was added to the upper chamber, and fluorescence in the lower chamber was measured at the indicated time points using a Clariostar plate reader (BMG Labtech). For cancer cell transmigration, HUVEC monolayers were pre-treated with exosomes and seeded with GFP-expressing Panc-1 cells on 8 µm pore inserts. Transmigrated cells in the lower chamber were counted 24 h later using a fluorescence microscope (Olympus, Tokyo, Japan) and ImageJ software.

### TEER measurement

The integrity and permeability of the EC monolayer were evaluated by measuring TEER. HUVECs were seeded on Transwell inserts (4 µm pore size) and treated with exosomes (1.8 × 10¹¹ particles/mL) for 3 days to generate a monolayer. TEER was measured using an Epithelial Volt/Ohm Meter (EVOM2; World Precision Instruments). TEER values (Ω/cm^2^) were compared with those from non-exosome-treated HUVEC controls. Measurements were performed in three independent experiments.

### IF

IF was performed as previously described [56]. Samples were fixed in 4% formaldehyde diluted in PBS for 15 min, blocked with 5% goat serum for 1 h at room temperature, and incubated overnight at 4 °C with primary antibodies. After washing, slides were incubated with fluorophore-conjugated secondary antibodies for 1 h, washed and mounted with Moviol®. Images were captured using a confocal microscope (Olympus).

### Exosomes labeling with cell membrane dye

Exosomes were labeled as previously described [58, 59]. Isolated exosomes (3.6 × 10^12^ Particles) were diluted to 500 μL with PBS (10 mM, pH 7.4) and mixed with 2 μL of DiR cell membrane dye (10 mM; Invitrogen). The mixture of exosomes and dye was incubated in the dark at 4 °C for 30 min, diluted to 10 mL with PBS, and centrifuged at 100 000 × *g* for 90 min to remove excess dye. Labeled exosomes were resuspended in 200 μL PBS.

### Exosome treatment in mice

BALB/c nude (nu/nu) mice received 1.8 × 10^11^ DiR labeled exosomes *via* tail vein injection. Organs were collected 24 h later and imaged with an IVIS Lumina system (Xenogen Corporation, Hopkinton, MA, USA). Lung sections were stained with CD31 antibody to assess exosome uptake by ECs. To evaluate exosomes on altering lung EC function, mice were injected with 1.8 × 10^11^ exosomes every 3 days for 3 weeks. Lung ECs were isolated by flow cytometry for molecular and phenotypic analysis. To further evaluate cancer cell extravasation, mice were pretreated with exosomes every 3 days for 2 weeks, followed by intravenous injection of 5 × 10^4^ GFP expressing Panc-1 cells. Exosomes treatment continued every 3 days for an additional 2 weeks before analysis. Mice were purchased from Vital River Laboratory Animal Technology Co.Ltd (Beijing, China) and maintained in a pathogen-free facility at Nankai University. All animal experiments were approved by the Nankai University Animal Care and Use Committee (Approval No. 2021-SYDWLL-000060) and handled according to the Nankai University Animal Welfare Guidelines.

### Mouse lung EC sorting by flow cytometry

Lung ECs were isolated from Balb/c mice after 3 weeks exosomes treatment. Single cell suspensions were prepared by collagenase digestion as previously described [60, 61]. Briefly, lungs were minced and digested in collagenase at 37 °C for 1 h. The digest was neutralized with culture medium, homogenized by passage through a 19.5-gauge needle, filtered through a 70-μm strainer to obatin single cell suspension. Single cells were stained with CD45 and CD31 antibodies. ECs were identified as CD31^+^ and CD45^−^ population and sorted using the FACSAria II. The isolated ECs were analyzed by qPCR and Western blot.

### H&E staining

H&E staining was conducted according to standard procedures [56]. Briefly, tissue sections were deparaffinized by 2 incubations in xylene (5 min each), followed by rehydration through a graded ethanol series (100%, 90%, 80%, 70% ethanol, 5 min each), and 2 washes in distilled water (2 min each). Sections were then stained with hematoxylin solution for 2 min and rinsed under running tap water for 10 min. Subsequently, sections were stained with eosin solution for 30 s to 2 min. Thereafter, sections were dehydrated through a reverse ethanol gradient (70%, 80%, 90%, and 100% ethanol, 10 s each) and cleared with 2 changes of xylene (5 min each). Finally, sections were mounted with a resinous mounting medium.

### Tissue TEM analysis

Lung tissues were subjected to TEM analysis following a standard protocol [62]. Briefly, samples were fixed with 2.5% glutaraldehyde for 3 h and post-fixed in 1% osmium tetroxide for 2 h. Tissues were dehydrated through a gradient of ethanol to acetone (50%, 70%, 80%, 90%, 95% and twice in 100% ethanol, followed by twice in 100% acetone) and embedded using an SPI Chem SPI-Pon^TM^ 812 Kit (SPI Supplies, West Chester, PA, USA). Ultrathin sections were obtained with a Leica EM UC7 ultramicrotome (Leica Microsystems, Wetzlar, Germany) and stained with uranyl acetate and lead nitrate. Images were acquired using a Hitachi HT7700 TEM (Hitachi, Tokyo, Japan) operating at 80 kV.

### Orthotopic xenograft mouse model of PAAD

An orthotopic xenograft model of PAAD was established as described previously [63]. Briefly, Nude mice were anesthesia by pentobarbital sodium (Sigma-Aldrich, St. Louis, MO, USA) and placed on a surgical pad. The left rib and abdomen area were cleaned with iodophor. The spleen and attached pancreas were exteriorized from a 2 mm subcostal incision on the left made by sterilized surgical scissors. A slip knot was loosely tied around the tail of the pancreas (white portion), taking care not to constrict the tissue tightly. A suspension of 5 × 10^5^ Aspc-1 cells in 30 μL PBS was injected into the pancreas through the slip knot. The knot was then tightened with controlled force. The spleen and pancreas were returned to the abdominal cavity. The incision was closed in one layer using 5-0 PDS-II surgical absorbable sutures (Ethicon, Inc., Somerville, NJ, USA).

### Statistical analysis

All statistical analyses were conducted using GraphPad Prism 7 for Windows (GraphPad Software, San Diego, CA, USA). Each independent experiment was repeated three times, and the results are presented as mean ± SEM. Statistical analyses used either a two-tailed Student’s *t* test or one-way analysis of variance (ANOVA). Animal experiments were conducted with three technical replicates, each comprising 3–6 mice per group. A *p*-value < 0.05 was considered statistically significant.

## Supplementary information

Supplementary figures and tables

Supplementary full blots

## Data Availability

All data generated in this study are available upon reasonable request from the corresponding author. Exosomal miRNA sequencing data analyzed in this study were obtained from Gene Expression Omnibus (GEO) (https://www.ncbi.nlm.nih.gov/geo/) under accession number GSE166799.
