# Supplementary material for: Exosomal miR-92a-3p promotes pancreatic cancer cells' extravasation by inducing vascular permeability through inhibition of DAB2IP
Source: Cell Death Dis. 2026 Apr 8;17(1):489. doi: 10.1038/s41419-026-08719-9 (PMC13187007; doi:10.1038/s41419-026-08719-9)
Supplement: Supplementary file 1 — Supplementary figures and tables [file 41419_2026_8719_MOESM1_ESM.pdf]

Supplementary data

Exosomal miR-92a-3p promotes pancreatic cancer cells extravasation by inducing vascular permeability through inhibition of DAB2IP

Luhan Li[1,2,#], Yanyan Cui[3,#], Miao Zhang[4], Tianyu Shen[2], Dekun Wang[2], Xue Mi[2], Yuying Zhang[2], Xiaoyue Tan[2], Alejandro Vaquero[5], Thomas Braun[6], Jihui Hao[7], Alessandro Ianni[5,6], Chunyang Jiang[1,*], Shijing Yue[1,2,*]

[1] Department of Thoracic Surgery, Tianjin Union Medical Center, The First Affiliated Hospital of Nankai University, Nankai University, Tianjin, 300121, China.

[2] School of Medicine, State Key Laboratory of Medicinal Chemical Biology, Nankai University, 94 Weijin Road, Tianjin, 300071, China.

[3] The Affiliated Hospital of Chifeng University, Chifeng, Inner Mongolia, 024005, China.

[4] Department of Oncology, Tianjin Union Medical Center, The First Affiliated Hospital of Nankai University, Nankai University, Tianjin, 300121, China.

[5] Chromatin Biology Laboratory, Josep Carreras Leukaemia Research Institute (IJC), Ctra de Can Ruti, Camí de les Escoles s/n, Badalona, Barcelona, Catalonia, 08916, Spain.

[6] Department of Cardiac Development and Remodeling, Max-Planck-Institute for Heart and Lung Research, Bad Nauheim, 61231, Germany.

[7] Tianjin Medical University Cancer Institute and Hospital, National Clinical Research Center for Cancer, Key Laboratory of Cancer Prevention and Therapy, Tianjin's Clinical Research Center for Cancer, Department of Pancreatic Cancer, Tianjin, 300060, China.

[#] The authors contributed equally to this work.

*Correspondence: Chunyang Jiang, E-mail: chunyangjiang@126.com and Shijing Yue, E-mail: shijingyue@nankai.edu.cn.

Content

1 Materials and methods..3
1.1 Construction of DAB2IP expressing and knodckdown vectors........................3
1.2 Generation of DAB2IP expressing and knodckdown cell lines........................3
1.3 Immunoprecipitation (IP)..3
2 Supplementary results and tables..4
2.1 Particle size and the miRNA analysis of plasma-derived exosomes................4
2.2 Strategy for miR-92A knockout (KO)...5
2.3 Characterization of exosomes derived from cell culture supernant..................6
2.4 DAB2IP regulates PI3K-AKT pathway in ECs..7
2.5 Exosomes enriched miR-92a-3p enhance cancer cell extravasation.................8
2.6 Patients and healthy donors information...8
2.7 Primer information..11
2.8 Antibody information..13
References...14

1 Materials and methods

1.1 Construction of DAB2IP expressing and knodckdown vectors

DAB2IP expressing and knodckdown HUVECs were generated as previously described (1). Human DAB2IP cDNA was obtained via reverse transcription of mRNA isolated from HUVECs. The DAB2IP cDNA was inserted into the lentiviral expression vector pLV-EF1α-MCS-IRES-Bsd/puro (Biosettia, San Diego, CA, USA). The empty vector generated lentiviral was used as the control. Primer sequences for DAB2IP cDNA cloning are listed in **Table. S8**. DAB2IP knodckdown is performed with the infection of lentiviral particles containing gene-targeting shRNAs. DAB2IP specific shRNAs was inserted into the lentiviral expression vector pLV-H1-RNAi-Vector (Biosettia, San Diego, CA, USA). Oligonucleotide sequences of shRNAs used in this study are listed in **Tables. S8**, respectively.

1.2 Generation of DAB2IP expressing and knodckdown cell lines

Lentiviral particles production was performed as previously described (2). Briefly, 6 x 10^6 HEK293T cells were seeded in a 10-cm dish and co-transfected with 9 μg of pLV-EF1α-MCS-IRES-puro, pLV-EF1α-DAB2IP-IRES-puro vector, or DAB2IP-shRNA containing pLV-H1-RNAi-Vector together with packaging and envelope vectors 4.5 μg pMDLg/pRRE, 1.8 μg pRSV-REV, 2.7 μg pCMV-VSV-G (Addgene) using Lipofectamine 3000 (Invitrogen Inc., Carlsbad, CA, USA). Lentiviral particles were collected at 48 h post-transfection and filtered through a 0.45 μm membrane. For HUVECs, or PAAD cells infection, lentiviral particles were diluted 1:2 in fresh medium and supplemented with 8 μg/mL polybrene and incubated. Cell culture medium was changed for an additional 48 h period. Infected cells were selected using puromycin (1 μg/mL). All the viral experiments were performed in a biological safety cabinet.

1.3 Immunoprecipitation (IP)

IP was performed following standard protocols as previously described (1). Briefly, cell lysates were collected in IP buffer (50 mM Tris-HCl; pH 7.4, 150 mM NaCl, 0.1% NP-40, 5 mM EDTA) supplemented with protease inhibitor cocktail (Sigma-Aldrich) and cleared by centrifugation. Protein lysates were incubated with specific antibodies as indicated or with non-immune immunoglobulin (IgG; negative control) and with G-agarose beads overnight on a rotating wheel. Next day, the beads were washed in ice-cold IP buffer, resuspended in 2x western blot loading buffer (65 mM Tris-HCl pH 6.8, 25% glycerol, 2% SDS, 0.01 % bromophenol blue, 50 mM DTT) and boiled at 95 °C for 5 min prior to western blot analysis.

2 Supplementary results and tables

2.1 Particle size and the miRNA analysis of plasma-derived exosomes

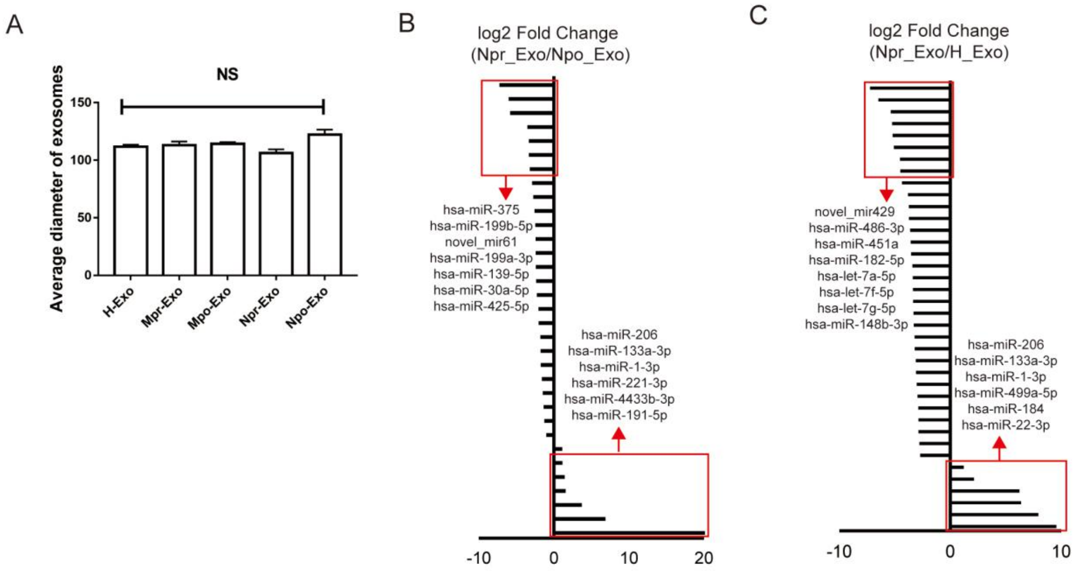

Figure. S1 Diameter and miRNA sequencing of PaCa patient-derived exosomes

A. Average diameter of exosomes derived from the plasma of PaCa patients (Mpr-Exo: metastatic pre-surgery; Mpo-Exo: metastatic post-surgery; Npr-Exo: non-metastatic pre-surgery, Npo-Exo: non-metastatic post-surgery) or healthy controls (H-Exo) as analyzed by Nanoparticle Tracking Analysis (NTA) device (NanoSight). one-way ANOVA, means ± SEM, n=3, NS $P \geq 0.05$. **B-C.** Histograms showing the log2 fold change in expression of indicated miRNAs in exosomes derived from patients of different groups, as determined by miRNA sequencing. H: Healthy; Npr: Non-metastatic pre-surgery; Npo: Non-metastatic post-surgery.

2.2 Strategy for miR-92A knockout (KO)

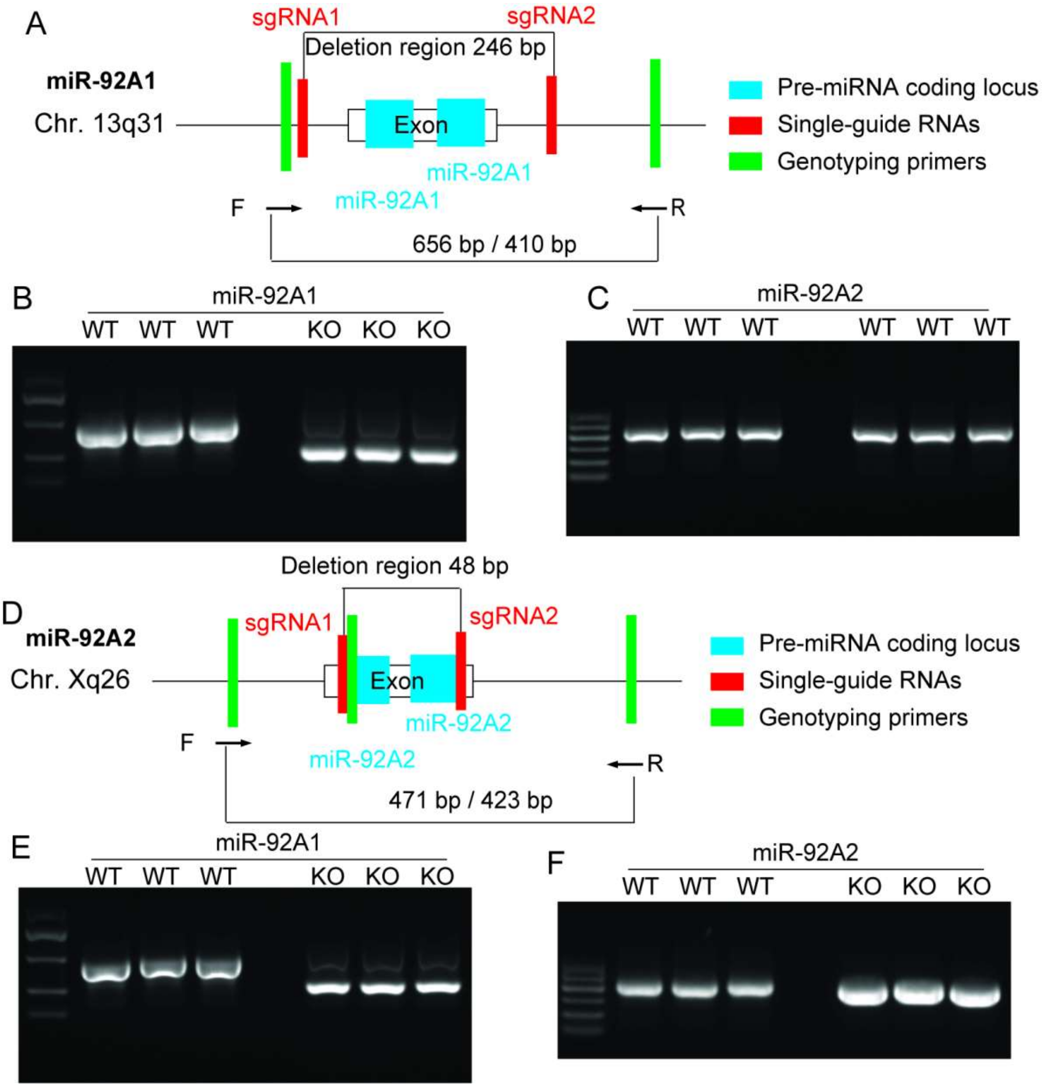

Figure. S2 Strategy for miR-92A KO and genotyping validation

A. Strategy of miR-92A1 with the pre-miRNA coding locus at Chr. 13q31. **B.** Genotyping validation of miR-92A1 KO by PCR. **C.** Genotyping validation of the wild type of miR-92A2 by PCR. **D**. Strategy of miR-92A2 with the pre-miRNA coding locus at Chr. Xq26. **E.** Genotyping validation of miR-92A1 KO by PCR. **F.** Genotyping validation of miR-92A2 KO by PCR.

2.3 Characterization of exosomes derived from cell culture supernant

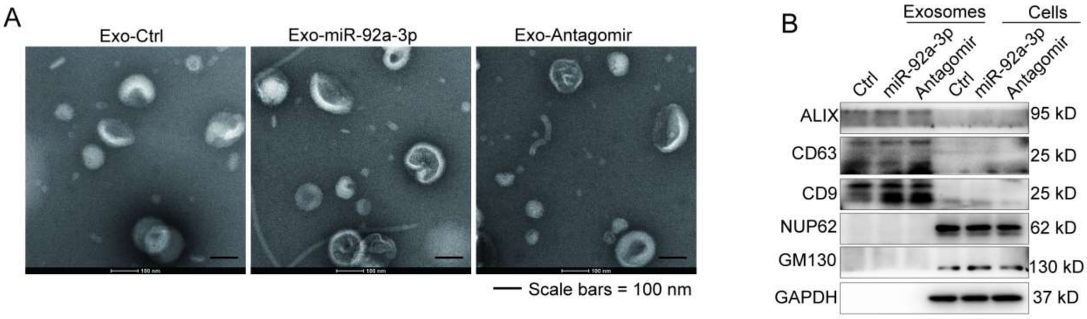

Figure. S3 Characterization of PaCa cells-derived exosomes

A. TEM analysis of exosomes isolated from Panc-1 cells transfected with miR-92a-3p, Antagomir, and control miRNAs demonstrating proper size and morphology. **B.** Western blot analysis the expression of exosomal (ALIX, CD63, and CD9), nuclear (NUP62) and Golgi (GM130) markers in exosomes and whole cell lysates derived from Panc-1 cells transfected with miR-92a-3p, Antagomir, and control miRNAs.

2.4 Exosomal miR-92a-3p promote EC permeability

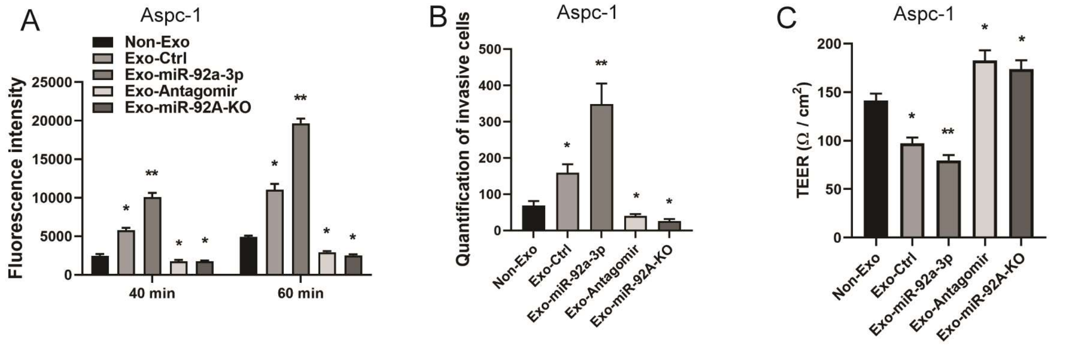

Figure. S4 Aspc-1 cell-derived exosomes enriched in miR-92a-3p promote EC permeability *in vitro*.

A. Quantification of fluorescence in the medium placed in the lower chamber of the rhodamine-dextran penetrate through the HUVEC monolayer pre-treated with Aspc-1 cell-derived exosomes. one-way ANOVA, means ± SEM, n = 3, *P < 0.05, **P < 0.01. **B.** Quantification of invasive Panc-1 cells transmigrated on HUVEC monolayer pre-treated with Aspc-1 cell-derived exosomes. one-way ANOVA, means ± SEM, n = 3, *P < 0.05, **P < 0.01. **C.** Quantification of TEER value of HUVEC monolayers. one-way ANOVA, means ± SEM, n = 3, *P < 0.05, **P < 0.01.

2.4 DAB2IP regulates PI3K-AKT pathway in ECs

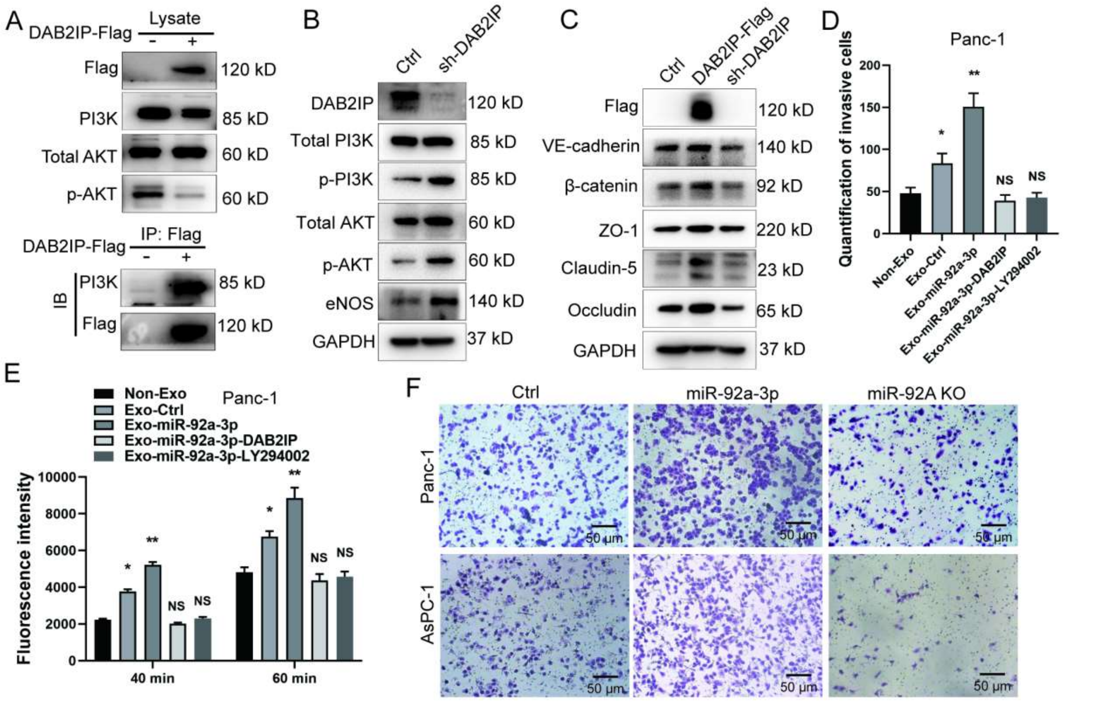

Figure. S5 DAB2IP inhibits the activation of PI3K-AKT pathway and induces cell-cell junction formation in HUVECs

A. Coupled immunoprecipitation (anti-Flag antibody) and western blot analysis (Anti-PI3K and anti-Flag antibodies) of cell lysates of HUVEC transfected with Flag-tagged DAB2IP as indicated. **B.** Western blot analysis of indicated markers in stable scrambled (Ctrl) and DAB2IP knockdown (sh-DAB2IP) HUVECs demonstrating increased activation (phosphorylation; p-) of the AKT-PI3K pathway in DAB2IP knockdown cells **C.** Western blot analysis of expression of indicated intracellular junction markers in HUVECs expressing Flag-tagged DAB2IP or knockdown DAB2IP by shRNA. **D.** Quantification of invasive Panc-1 cells transmigrated on HUVEC monolayer pre-treated with Panc-1 cell-derived exosomes for overexpressing DAB2IP rescue assay and PI3K inhibitor LY294002 treatment. one-way ANOVA, means ± SEM, $n = 3$, NS $P \geq 0.05$, $^*P < 0.05$, $^{**}P < 0.01$. **E.** Quantification of fluorescence in the medium placed in the lower chamber of the rhodamine-dextran penetrate through the HUVEC monolayer pre-treated with Panc-1 cell-derived exosomes for overexpressing DAB2IP rescue assay and PI3K inhibitor LY294002 treatment. one-way ANOVA, means ± SEM, $n = 3$, NS $P \geq 0.05$, $^*P < 0.05$, $^{**}P < 0.01$. **F.** PAAD cell motility was assessed using Transwell assays with miR-92a-3p expressing and miR-92A KO.

2.5 Exosomes enriched miR-92a-3p enhance cancer cell extravasation

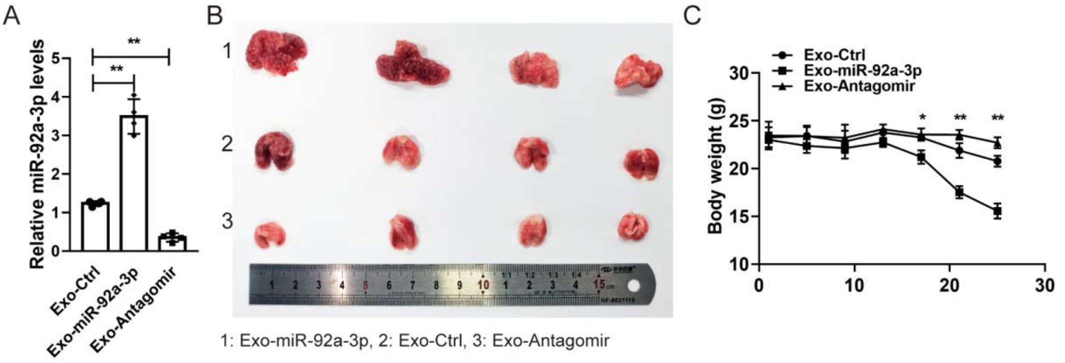

Figure. S6 Exosomal miR-92a-3p promotes PaCa cancer metastasis in the lung

A. Relative miRNA-92a-3p levels in lung tissues of mice intravenously injected with PaCa cells and pre-treated and post-treated with exosomes as described in main figure 5. one-way ANOVA, means ± SEM, n = 4, **P < 0.01. **B.** Representative PaCa lung metastasis in mice treated as described in main figure 5. **C.** Body weight variations in nude mice treated as described in main figure 5. one-way ANOVA, means ± SEM, n = 4, *P < 0.05, **P < 0.01.

2.6 Patients and healthy donors information

Table. S1 Clinical information of PaCa patients

No.	Non-metastasis（9）	Lung metastasis（12）	Liver metastasis（9）	Lymph node metastasis（7）	Intraperitoneal metastasis (7)	Gender	Age
1	N	P	P	P	N	F	62
2	N	P	N	P	N	F	45
3	P	N	N	N	N	F	68
4	P	N	N	N	N	M	46
5	N	P	P	P	P	F	66
6	P	N	N	N	N	M	58
7	N	N	P	N	N	F	60
8	N	P	N	P	P	F	55
9	P	N	N	N	N	M	54
10	N	P	P	N	N	M	43
11	N	P	P	N	P	M	65
12	N	P	N	P	N	F	65
13	N	P	P	N	P	M	55
14	P	N	N	N	N	F	58
15	P	N	N	N	N	M	47
16	P	N	N	N	N	F	44
17	N	P	P	N	P	M	48
18	N	P	N	P	P	F	66

19	P	N	N	N	N	M	46
20	N	N	N	P	P	M	46
21	N	P	P	N	N	M	62
22	P	N	N	N	N	M	68
23	N	P	P	N	N	M	65

P: Positive; **N**: Negative; **F**: Female; **M**: Male

Table S1. The clinical information of PaCa patients.

Blood samples were collected for exosomes isolation from the 23 PAAD patients. The patients were diagnosised as PAAD with or without metastsis.

Table. S2 Correlation of lung metastasis and liver, lymph node, or intraperitoneal metastasis

		Lung metastasis		*P*.value
		N	P	
Lymph node metastasis	N	9	7	0.442
	P	2	5	
Liver metastasis	N	11	0	0.000*
	P	0	12	
Intraperitoneal metastasis	N	10	4	0.016*
	P	1	8	

P: Positive; **N**: Negative; *P* value with were calculated by continuous calibration chi-square test.

Table. S2 Correlation of lung metastasis and liver, lymph node, or intraperitoneal metastasis

Correlation analysis was performed between lung metastasis and liver, lymph node, or intraperitoneal metastasis.

Table. S3 Correlation of liver metastasis and lung, lymph node, or intraperitoneal metastasis

		Liver metastasis		*P*.value
		N	P	
Lung metastasis	N	11	0	0.000*
	P	0	12	
Lymph node metastasis	N	9	7	0.442
	P	2	5	
Intraperitoneal metastasis	N	10	1	0.016*
	P	4	8	

P: Positive; **N**: Negative; *P* value with were calculated by continuous calibration chi-square test.

Table. S3 Correlation of liver metastasis and lung, lymph node, or intraperitoneal metastasis

Correlation analysis was performed between liver metastasis and lung, lymph node, or intraperitoneal metastasis.

Table. S4 Correlation of lymph node metastasis and lung, liver, or intraperitoneal metastasis

		Lymph node metastasis		*P*.value
		N	P	
Liver metastasis	N	9	7	0.442
	P	2	5	
Lung metastasis	N	9	7	0.442
	P	2	5	
Intraperitoneal metastasis	N	13	3	0.010*
	P	1	6	

P: Positive; **N**: Negative; *P* value with were calculated by continuous calibration chi-square test.

Table. S4 Correlation of lymph node metastasis and lung, liver, or intraperitoneal metastasis

Correlation analysis was performed between lymph node metastasis and lung, liver, or intraperitoneal metastasis.

Table. S5 Correlation of intraperitoneal metastasis and lung, liver, or lymph node metastasis

		Intraperitoneal metastasis		*P*.value
		N	P	
Lung metastasis	N	13	3	0.010*
	P	1	6	
Liver metastasis	N	10	1	0.016*
	P	4	8	
Lymph node metastasis	N	10	1	0.016*
	P	4	8	

P: Positive; **N**: Negative; *P* value with were calculated by continuous calibration chi-square test.

Table. S5 Correlation of intraperitoneal metastasis and lung, liver, or lymph node metastasis

Correlation analysis was performed between intraperitoneal metastasis and lung, liver, or lymph node metastasis.

Table. S6 General information of healthy donors

No.	Gender	Age
1	M	24
2	M	25
3	M	28
4	F	36
5	F	36
6	M	45
7	F	50
8	M	44
9	F	54
10	M	43
11	F	35
12	F	45
13	F	55
14	M	50
15	M	57
16	M	24
17	F	48
18	M	56
19	F	46
20	M	36

F: Female; **M**: Male

Table. S6 The general information of healthy donors.

Blood samples were collected for exosomes isolation from the 20 healthy donors.

2.7 Primer information

Table. S7 Oligonucleotide sequences used for miRNA lentiviral constructs

Gene	Target sequence (5'-3')
miR-92a-3p	5'-TATTGCACTTGTCCCGGCCTGT-3'
Negative control	5'-TTCTCCGAACGTGTCACGT-3'
Antagomir of miR-92a-3p	5'-ACAGGCCGGGACAAGTGCAATA-3'

Table. S7 The sequences of oligonucleotide for lentiviral constructs.

The lentiviral constructs were generated with the oligonucleotide to express miRNA control, miR-92a-3p, and antagomir of miR-92a-3p.

Table. S8 Primers used for DAB2IP expressing and knockdown shRNA

Gene	Sequence (5'-3')
DAB2IP-sh1	5'-AAAAGGGATAAGTGGATGGAGAATTGGATCCAATTCT CCATCCACTTATCCC-3'
DAB2IP-sh2	5'-AAAAGGGAGACCGACAAGAAGAATTGGATCCAATTC TTCTTGTCGGTCTCCC-3'

DAB2IP-sh3	5-'AAAAGCAAGATCATCAACTCCTATTGGATCCAATAGG AGTTGATGATCTTGC-3'
DAB2IP-F	5'-GCTCTAGAATGGAGCCCGACTCCCTTC-3'
DAB2IP-R	5'-GCGGATCCTCACTTATCGTCGTCATCCTTGTAATCATG CATACTCTCTTTCAGC-3'

Table. S8 The sequences of primers for DAB2IP expressing and knockdown.
The constructs were generated with the primers to clone the CDS DAB2IP for expression and the shRNA expression for DAB2IP knockdown.

Table. S9 Sequence for single-guide RNAs used for *miR-92A1/2* deletion

Gene	Sequence information (5'-3')
miR-92a1 sgRNA1 F	5'-CACCGGTTACTGAACACTGTTCTAT-3'
miR-92a1 sgRNA1 R	5'-AAACATAGAACAGTGTTCAGTAACC-3'
miR-92a1 sgRNA2 F	5'-CACCGGTATCTTGTACATTTAACAG-3'
miR-92a1 sgRNA2 R	5'-AAACCTGTTAAATGTACAAGATACC-3'
miR-92a2 sgRNA1 F	5'-CACCGATGCAACAAATCCCCACCCA-3'
miR-92a2 sgRNA1 R	5'-AAACTGGGTGGGGATTTGTTGCATC-3'
miR-92a2 sgRNA2 F	5'-CACCGATAAAGTATTGCACTTGTCC-3'
miR-92a2 sgRNA2 R	5'-AAACGGACAAGTGCAATACTTTATC-3'

Table. S9 The sequences of sgRNA for *miR-92A1/2* deletion.
The constructs of CRISPR/Cas9 were generated with the sgRNA for *miR-92A1/2* knockout.

Table. S10 Sequence of genotyping primers used for *miR-92A1/2* deletion

Gene	Sequence information (5'-3')
miR-92a1 genotyping F	5'-CCTGTCGCCCAATCAAACTG-3'
miR-92a1 genotyping R	5'-CCTGGAATAACACTAACTCC-3'
miR-92a2 genotyping F	5'-GGGCACTTCCAGTACTCTTG-3'
miR-92a2 genotyping R	5'-GGAATGTCGCCAACAAAGGTC-3'

Table. S10 The sequences of primers for *miR-92A1/2* knockout genotyping.
The primers were used for PCR of *miR-92A1/2* knockout cells genotyping.

Table. S11 Primers used for miRNA stem-loop RT-qPCR

Gene	Sequence (5'-3')
miR-92a-3p-Stem loop	5′-GTCGTATCCAGTGCAGGGTCCGAGGTATTC GCACTGGATACGACACAGGC-3′
miR-92a-3p-F	5′-GCGTATTGCACTTGTCCCG -3′
Universal reverse primer	5′-CTGGTGCAGGGTCCGAGGTAT-3′
18S-F	5′-GTAACCCGTTGAACCCCATT-3′
18S-R	5′-CCATCCAATCGGTAGTAGCG-3′
U43-F	5′-CACAGATGATGAACTTATTGACG-3′
U43-R	5′-CAGAACGTGACAATCAGCAC-3′
U6-F	5′-GCTCGCTTCGGCAGCACATATACTAA-3′

U6-R	5′-ACGAATTTGCGTGTCATCCTTGCG-3′

Table. S11 The sequences of primers for miRNA stem-loop RT-qPCR.
The primers were used for stem-loop RT-qPCR of miR-92a-3p and internal controls.

Table. S12 Primers used for target gene RT-qPCR analysis

Gene	Sequence (5’-3’)
DAB2IP-F	5′-CTGAGCGGGATAAGTGGATGG-3′
DAB2IP-R	5′-AAACATTGTCCGTCTTGAGCTT-3′
VE-Cadherin-F	5′- CAGCCCAAAGTGTGTGAGAA -3′
VE-Cadherin-R	5′- CGGTCAAACTGCCCATACTT -3′
ZO-1-F	5′-CAACATACAGTGACGCTTCACA-3′
ZO-1-R	5′-CACTATTGACGTTTCCCCACTC-3′
GAPDH-F	5′-GGAGCGAGATCCCTCCAAAAT -3′
GAPDH-R	5′-GGCTGTTGTCATACTTCTCATGG-3′

Table. S12 The sequences of primers for RT-qPCR.
The primers were used for RT-qPCR of genes and internal control.

Table. S13 Primers used for generation of luciferase constructs

Gene	Sequence (5’-3’)
DAB2IP-WT-F	5′-CGGGTACCGTAAGTATGTGAAAAGGA-3′
DAB2IP-WT-R	5′-CGGCTAGCATAGCTAAATAAATCTTTA-3′
DAB2IP-AS-F	5′-CGGGTACCTGGCTGCGTACTTTCTGTACATTT-3′
DAB2IP-AS-R	5′-CGGCTAGCAGAAAGTACGCAGCCACACAGAGT-3′

Table. S13 The sequences of primers for generation of luciferase constructs.
The primers were used for generation of luciferase constructs.

2.8 Antibody information

Table. S14 List of antibodies used in this study

Antibody	Company	Cat. No.
β-Actin	ZSGB-BIO	TA-09
GAPDH	Abcam	Ab82633
ALIX	Santa Cruz	SC-99010
CD9	CST	13403S
CD63	Abcam	Ab193349
NUP62	Santa Cruz	SC-166870
GM130	Santa Cruz	SC-55591
VE-cadherin	CST	2158s
β-catenin	Biolegend	610253
ZO-1	Abcam	Ab216880
Claudin-5	Santa Cruz	SC-374221
Occludin	CST	91131S
eNOS	Santa Cruz	SC-1025
PI3K p85	CST	4292S

p-PI3K	Biolegend	610446
Total AKT	CST	4691S
p-AKT	CST	4060S
DAB2IP	Proteintech	23582-1-AP
Anti-Flag	Sigma-Aldrich	F2555
Anti-mouse	ZSGB-BIO	ZB-2305
Anti-rabbit	ZSGB-BIO	ZB-2301
Anti-rabbit	ZSGB-BIO	SP-9002

Table. S14 The antibodies were used in this study.

The information of antibodies used in this study.

References

1. Li J, Xu J, Li L, Ianni A, Kumari P, Liu S, et al. MGAT3-mediated glycosylation of tetraspanin CD82 at asparagine 157 suppresses ovarian cancer metastasis by inhibiting the integrin signaling pathway. Theranostics. 2020;10:6467-82.

2. Liu S, Wang H, Li J, Zhang J, Wu J, Li Y, et al. FZR1 as a novel biomarker for breast cancer neoadjuvant chemotherapy prediction. Cell Death Dis. 2020;11(9):804.
