# Supplementary material for: Exosomal miR-92a-3p promotes pancreatic cancer cells' extravasation by inducing vascular permeability through inhibition of DAB2IP
Source: Cell Death Dis. 2026 Apr 8;17(1):489. doi: 10.1038/s41419-026-08719-9 (PMC13187007; doi:10.1038/s41419-026-08719-9)

Supplementary full blots of this study

Exosomal miR-92a-3p promotes pancreatic cancer cells extravasation by inducing vascular permeability through inhibition of DAB2IP

Luhan Li[1,2,#], Yanyan Cui[3,#], Miao Zhang[4], Tianyu Shen[2], Dekun Wang[2], Xue Mi[2], Yuying Zhang[2], Xiaoyue Tan[2], Alejandro Vaquero[5], Thomas Braun[6], Jihui Hao[7], Alessandro Ianni[5,6], Chunyang Jiang[1,*], Shijing Yue[1,2,*]

[1] Department of Thoracic Surgery, Tianjin Union Medical Center, The First Affiliated Hospital of Nankai University, Nankai University, Tianjin, 300121, China.

[2] School of Medicine, State Key Laboratory of Medicinal Chemical Biology, Nankai University, 94 Weijin Road, Tianjin, 300071, China.

[3] The Affiliated Hospital of Chifeng University, Chifeng, Inner Mongolia, 024005, China.

[4] Department of Oncology, Tianjin Union Medical Center, The First Affiliated Hospital of Nankai University, Nankai University, Tianjin, 300121, China.

[5] Chromatin Biology Laboratory, Josep Carreras Leukaemia Research Institute (IJC), Ctra de Can Ruti, Camí de les Escoles s/n, Badalona, Barcelona, Catalonia, 08916, Spain.

[6] Department of Cardiac Development and Remodeling, Max-Planck-Institute for Heart and Lung Research, Bad Nauheim, 61231, Germany.

[7] Tianjin Medical University Cancer Institute and Hospital, National Clinical Research Center for Cancer, Key Laboratory of Cancer Prevention and Therapy, Tianjin's Clinical Research Center for Cancer, Department of Pancreatic Cancer, Tianjin, 300060, China.

[#] The authors contributed equally to this work.

*Correspondence: Chunyang Jiang, E-mail: chunyangjiang@126.com and Shijing Yue, E-mail: shijingyue@nankai.edu.cn.

1. Full blots of Figures

1.1 Full blots of Figure. 1

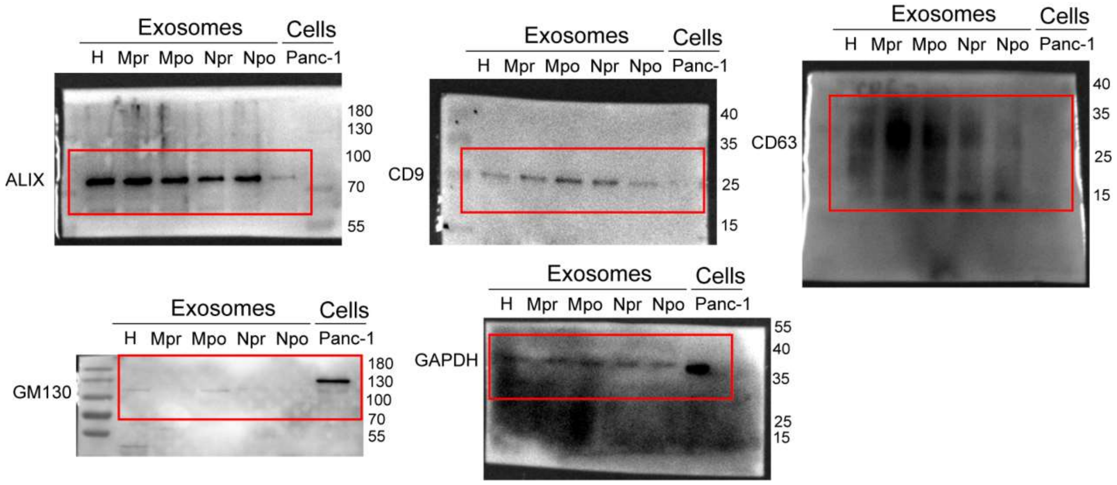

1.2 Full blots of Figure. 3

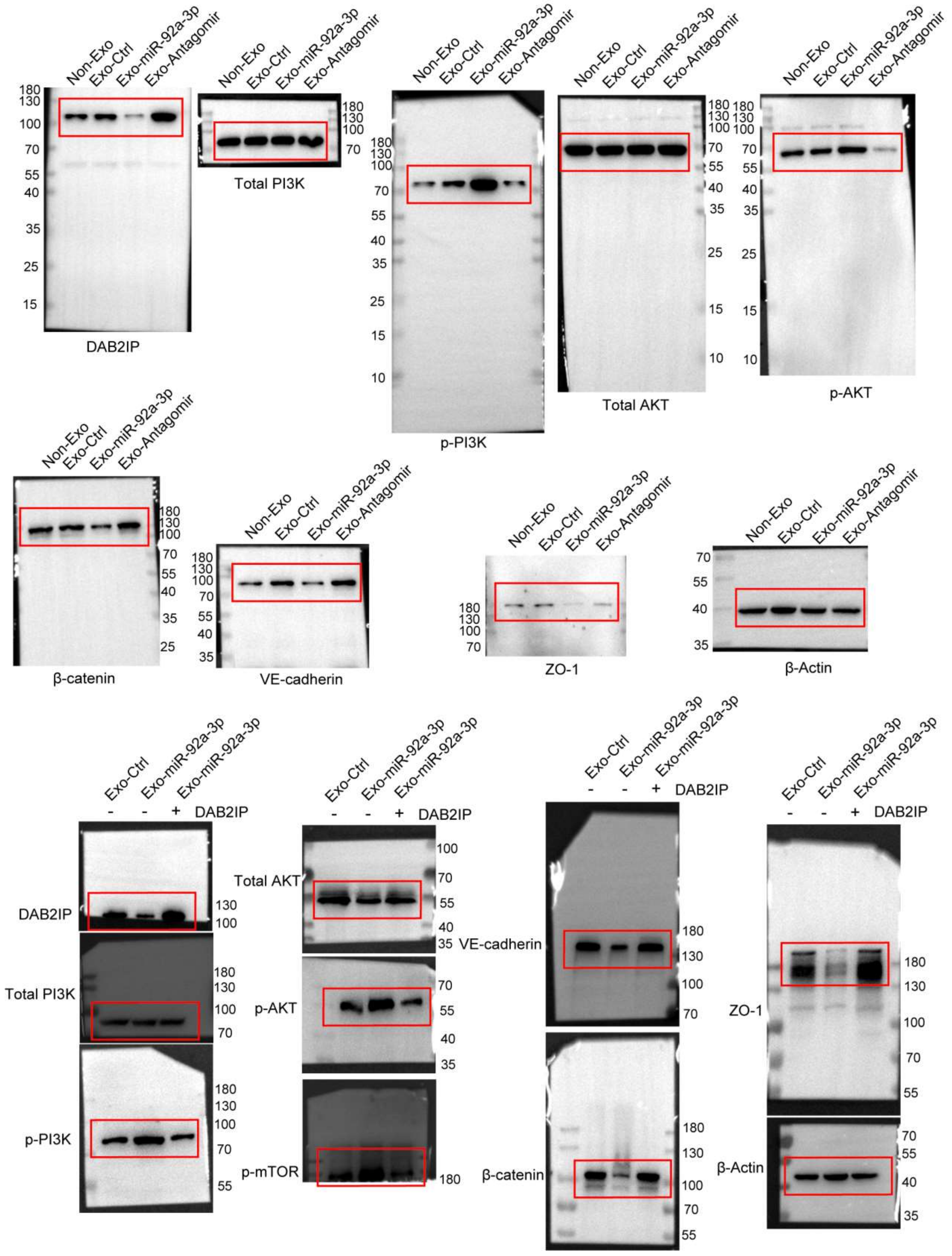

1.3 Full blots of Figure. 4

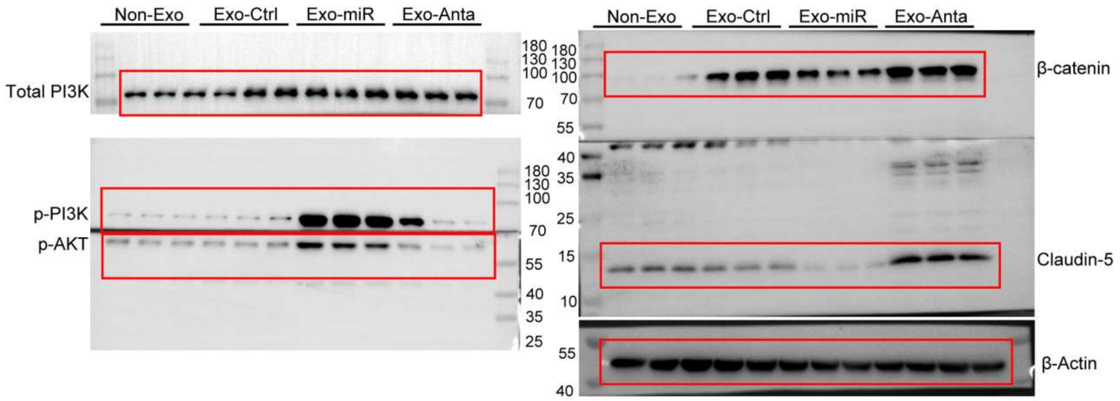

1.4 Full blots of Figure. 5

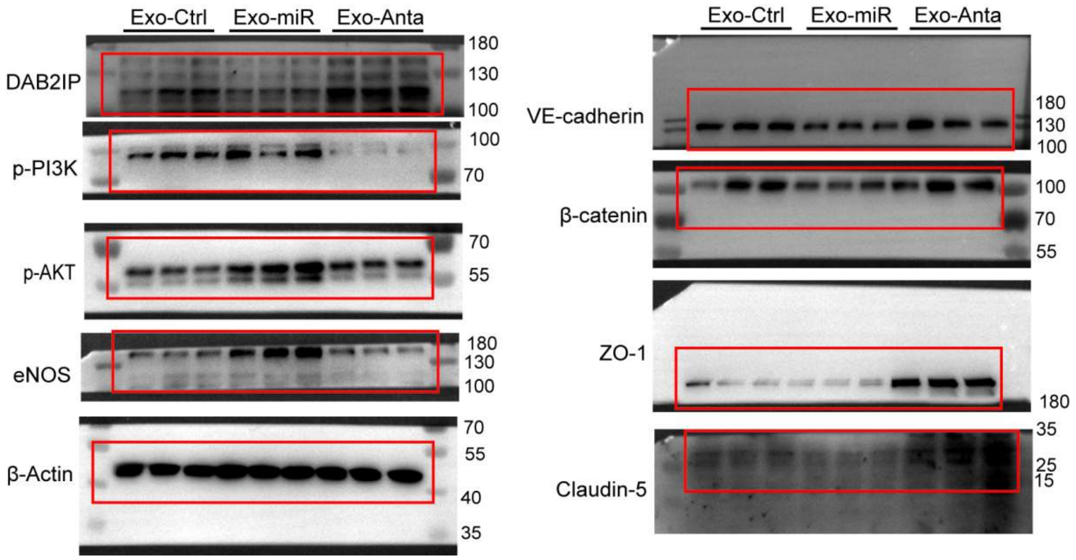

1.5 Full blots of Figure. 6

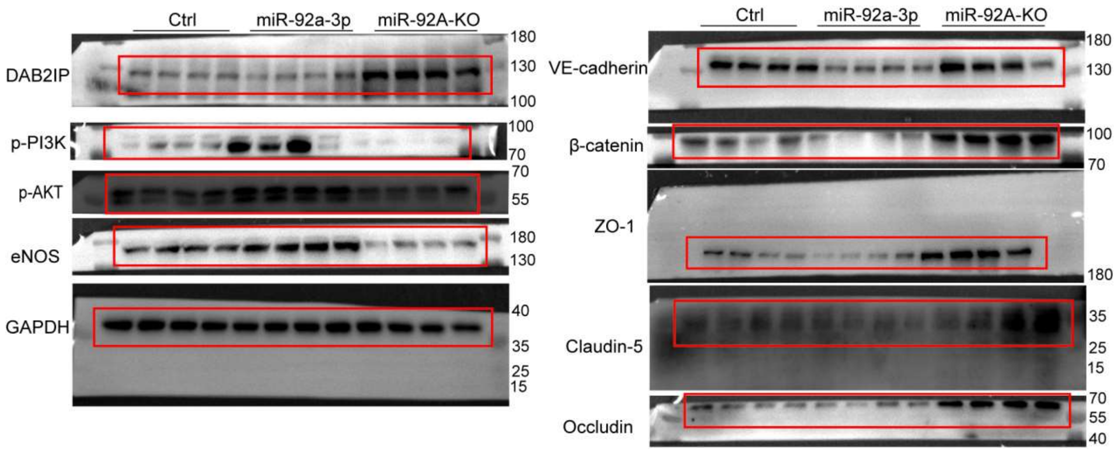

1.6 Full blots of Figure. S3

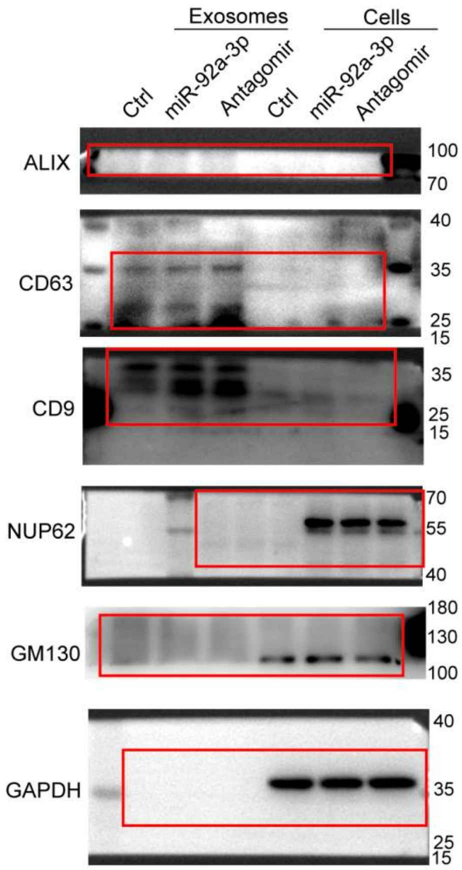

1.7 Full blots of Figure. S5

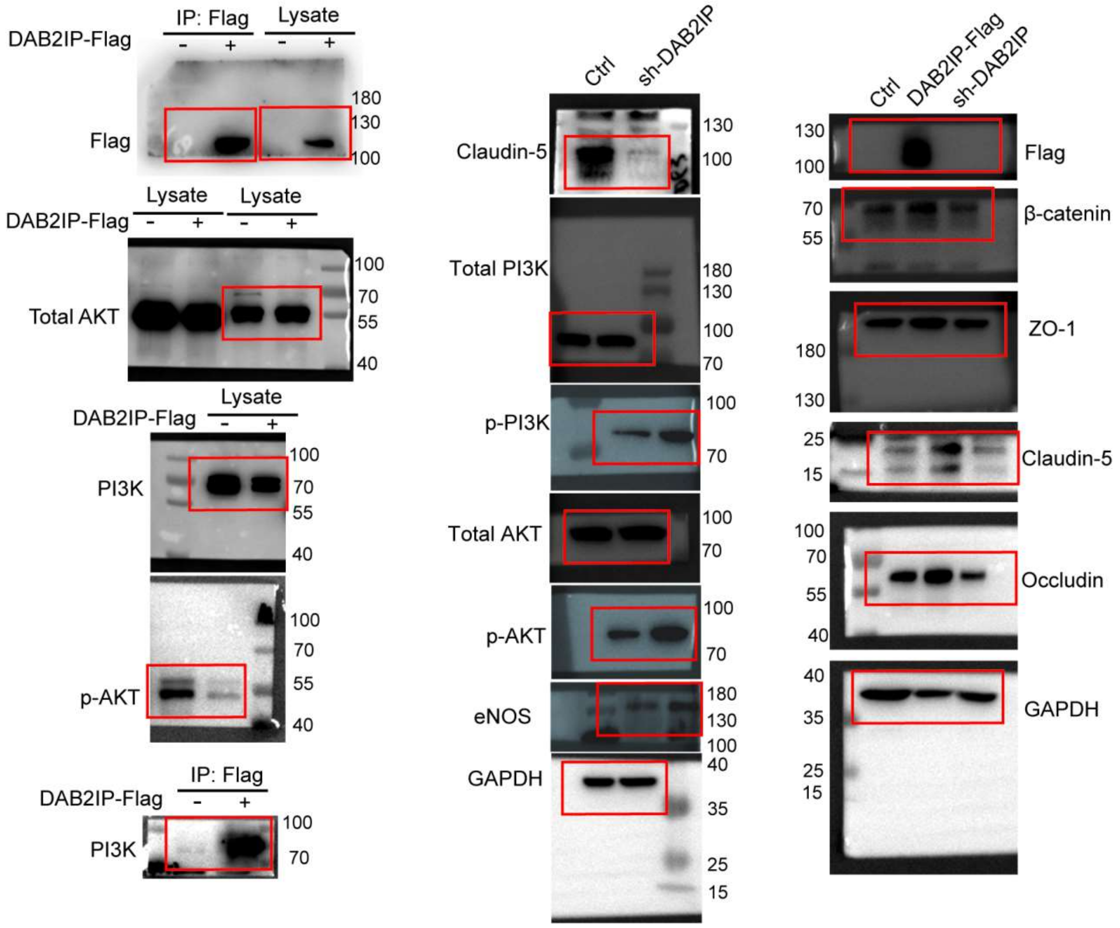

Supplement: Supplementary file 2 — Supplementary full blots [file 41419_2026_8719_MOESM2_ESM.pdf]
